# Synthesis of New 5′-Norcarbocyclic Aza/Deaza Purine Fleximers - Noncompetitive Inhibitors of *E.coli* Purine Nucleoside Phosphorylase

**DOI:** 10.3389/fchem.2022.867587

**Published:** 2022-05-04

**Authors:** Anastasia Khandazhinskaya, Ilja Fateev, Irina Konstantinova, Roman Esipov, Konstantin Polyakov, Katherine Seley-Radtke, Sergey Kochetkov, Elena Matyugina

**Affiliations:** ^1^ Engelhardt Institute of Molecular Biology of the Russian Academy of Sciences, Moscow, Russia; ^2^ Shemyakin-Ovchinnikov Institute of Bioorganic Chemistry, Russian Academy of Sciences, Moscow, Russia; ^3^ Department of Chemistry and Biochemistry, University of Maryland, Baltimore County, Baltimore, MD, United States

**Keywords:** fleximers, 5'-norcarbocyclic nucleoside analogs, inhibitor, purine nucleoside phosphorylase, pyrazole derivatives

## Abstract

A new series of flexible 5′-norcarbocyclic aza/deaza-purine nucleoside analogs were synthesized from 6-oxybicyclo[3.1.0.]hex-2-ene and pyrazole-containing fleximer analogs of heterocyclic bases using the Trost procedure. The compounds were evaluated as potential inhibitors of *E. coli* purine nucleoside phosphorylase. Analog **1-3** were found to be noncompetitive inhibitors with inhibition constants of 14–24 mM. From the data obtained, it can be assumed that the new 5′-norcarbocyclic nucleoside analogs interact with the active site of the PNP like natural heterocyclic bases. But at the same time the presence of a cyclopentyl moiety with 2′ and 3′ hydroxyls is necessary for the inhibitory properties, since compounds **8–10**, without those groups did not exhibit an inhibitory effect under the experimental conditions.

## Introduction

Purine nucleoside phosphorylase (PNP, EC 2.4.2.1) is a key enzyme involved in the metabolism of purine nucleosides, promoting the utilization of heterocyclic bases. PNP catalyzes the reversible phosphorolysis reaction of purine (deoxy)ribonucleosides to the corresponding bases and (deoxy)ribose-1-phosphate, thereby regulating the concentration of purines in the cell. The enzyme is a well-known biocatalyst in the synthesis of natural nucleosides and their analogs. The reaction facilitates a transfer of the pentafuranose residue from the donor nucleoside to a new heterocyclic base. Bacterial PNP has wide substrate specificity, and this property has found an applications in biotechnology approaches for the production of various biologically active natural and modified nucleosides on an industrial scale (Mikhailopulo, 2007; Mikhailopulo and Miroshnikov, 2011).

It was shown that different fleximer bases are substrates of PNP from *E. coli*, which was used for the chemo-enzymatic synthesis of the corresponding analogs of ribo- and 2′-deoxyribo-nucleosides (Vichier-Guerre et al., 2016; 2017; Vichier-Guerre et al., 2020). In our previous work pyrazole-containing fleximer analogs of heterocyclic bases were used as substrates for PNP (Khandazhinskaya et al., 2021). Despite the fact that the fleximer bases differ significantly from the natural substrates of the enzyme, by various aza/deaza modifications and the of the absence of the fused five and six-membered rings, they have proven to be effective substrates. Since interest in PNP research is associated not only with biotechnological uses, but also with the fact that the enzyme is a target in immunosuppressive and anticancer therapies, it was of interest to design PNP inhibitors based on fleximer analogs of pyrazole-containing heterocyclic bases. Most of the PNP inhibitors are structural analogs of nucleoside substrates, modified at the base and/or (deoxy)ribofuranose moieties, and also carrying an acyclic or heterocyclic residue as a carbohydrate component (Supplementary Figure S1, ) (Morris and Montgomery, 1998; Pogosian et al., 2011). Well-known *E. coli* PNP inhibitors such as aza/deaza purine nucleoside analog have inhibition constant values less than 10 µmol. The other inhibitor is 2-chloro-6-(3-phenyl-1-propoxy)purine with a *Ki* value of 1.4 µmol (Bzowska et al., 2000).

Based on previous experience with 5′-norcarbocyclic nucleoside analogs from our group, several of which proved to be effective inhibitors of various enzymes (Schneller, 2002; Matyugina et al., 2012), we decided to replace the carbohydrate fragment of previously synthesized ribonucleosides (Khandazhinskaya et al., 2021) with a carbocyclic residue and evaluate the new fleximer nucleoside analogs as potential inhibitors of purine nucleoside phosphorylase *E. coli*.

5′-Nor-nucleoside analogs belong to the class of carbocyclic nucleosides, in which the furanose oxygen atom has been replaced by a methylene group and the 5′-CH_2_ group is absent, thus the hydroxyl group is directly connected to C-4. Over the last several decades, carbocyclic nucleosides have exhibited potent and interesting biological activities (Seley-Radtke and Yates, 2018). The close similarity of their structure with the naturally occurring nucleosides allows them to act as substrates or inhibitors of various enzymes (Matyugina et al., 2012). The 5′-nor modification has been used as a possible way to reduce the cytotoxicity of carbocyclic nucleosides, which are efficiently recognized by cellular kinases and, in their phosphorylated forms, participate in key metabolic processes in the cell, competing with natural nucleosides. Without 5′-CH2 group, the phosphorylation of the 5′-norcarbocyclic nucleoside analogs does not occur (Schneller, 2002) thereby decreasing their cytotoxicity. At the same time, biological properties not associated with phosphorylation are retained (Matyugina et al., 2012). Herein the synthesis of several modified 5′-norcarbocyclic nucleoside analogs are described.

**FIGURE 1:**
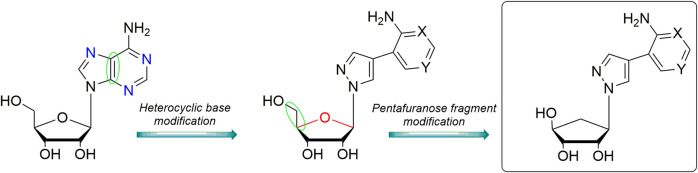
Design of 5′-norcarbocyclic aza/deaza-purine fleximers.

The newly designed compounds (Figure 1) contain a 5′-norcarbocyclic residue in place of the carbohydrate moiety and the heterocyclic base is 8-aza-7-deazapurine fleximer analog. A number of aza/deaza nucleoside analogs exhibit a wide variety of biological activities (Seley et al., 1997a; Seley et al., 1997b; Matyugina et al., 2021) including as PNP inhibitors (Timofeev et al., 2018). Carbocyclic deazapurine nucleoside analog have shown antiviral activity against herpes simplex virus, HIV-1, human cytomegalovirus, influenza A and hepatitis B viruses, norovirus, Ebola and measles viruses (Vittori et al., 2006; Matyugina et al., 2021).

Another structural modification which has proven effective was the development of the fleximers – nucleoside analogues wherein the purine base has been split into two separate heterocyclic fragments but remains connected by a single C-C bond (Seley et al., 2002; Seley et al., 2005; Peters H. et al., 2015; Ku and Seley-Radtke, 2018). Thus, the fleximers exhibit additional conformational freedom in order to maximize structural interactions in the active site of the target enzyme while maintaining the structural similarity with a normal nucleoside substrate or inhibitor necessary for recognition by the enzyme (Seley et al., 2005). Fleximers have exhibited a wide range of antiviral activities including filoviruses (Ebola and Marburg), coronaviruses (SARS, MERS and human coronaviruses) and flaviviruses (Dengue, Zika, Yellow fever) (Peters H. L. et al., 2015; Yates et al., 2017; Thames et al., 2020). Combining these structural modifications into one approach provided a series of 5′-norcarbocyclic aza/deazapurine fleximers that were then synthesized and evaluated their inhibitory properties against *E. coli* PNP.

## Materials and Methods

The reactions were performed with commercial reagents (Acros, Aldrich, and Fluka); anhydrous solvents were purified according to the standard procedures. Column chromatography was performed on Silica Gel 60 0.040–0.063 mm (Merck, Germany) columns, Dowex-50 (H^+^). Preparative liquid chromatography (PLC) was performed on Silica Gel 60 F_254_ with concentrating zone glass plates (Merck, Germany). Thin layer chromatography (TLC) was performed on Silica Gel 60 F_254_ aluminium-backed plates (Merck, Germany).

NMR spectra were recorded on a Bruker Avance III spectrometer (Bruker BioSpin, Rheinstetten, Germany) with an operating frequency of 300 MHz for ^1^H-NMR and 75.5 MHz for ^13^C-NMR in CDCl_3_, CD_3_OD or DMSO-d_6_. We designated the pyrazole fragment as A, and the pyridine or pyrimidine fragment as B for convenient correlation the signals in the spectra.

High resolution mass spectra (HRMS) were obtained on a Bruker Daltonics micrOTOF-Q II or maXis (Bruker, Germany) instruments using electrospray ionization (ESI). The measurements were acquired in a negative ion mode with the following parameters: interface capillary voltage—3700 V; mass range from *m*/*z* 50 to 3,000; external calibration (Electrospray Calibrant Solution, Fluka); nebulizer pressure—0.3 Bar; flow rate—3 μl/min; dry gas nitrogen (4.0 L/min); interface temperature was set at 180 or 190°C; or in a positive ion mode with the following parameters: interface capillary voltage 4500 V; mass range from m/z 50 to m/z 3,000 Da; external or internal calibration was done with Electrospray Calibrant Solution (Fluka, Switzerland), dry gas nitrogen (3.0 L/min); interface temperature was set at 180 C. A syringe injection was used.

The method used to produce recombinant *E. coli* phosphorylases was described earlier (Esipov et al., 2002, the enzyme is high stable in tested conditions (Lee, 2001).

### General Procedure for the Synthesis of Compounds 5–7 and 11, 12

Pyrazole-containing flexible bases (Khandazhinskaya et al., 2021) (0.3–0.5 mmol) were dissolved in DMF and re-evaporated 2 times. Then 6-oxybicyclo[3.1.0.]hex-2-ene (1.5 equiv.) in 2–3 ml THF and Pd(PPh_3_)_4_ (5 mol%) were added. The reaction mixture was stirred during 18 h and solvents were evaporated. The products were purified by column chromatography on silica gel to give compounds **5-7** in 68–79% yields and 11, 12 in 21 and 14% yields consequently (Characterisation of NMR spectra of compounds **5–7** and **11**, **12** see Supplementary Materials).

### General Procedure for the Synthesis of Compounds 8–10

Compounds **5–7** (1 equiv.) were dissolved in methanol (10 ml) and refluxed with K_2_CO_3_ (1.3 equiv.) during 36–48 h. The products were purified by column chromatography on silica gel (eluent chloroform: methanol (9:1)) to give compounds **8–10** in 63–86% yields.

1-(4′-Hydroxy-2′-cyclopenten-1′-yl)-4-(4-aminopyridin-3-yl)pyrazole (**8**). Pale yellow powder. Yield 86%. ^1^H NMR (300 MHz, CD_3_OD) δ: 8.06 (1H, s, H-2B), 8.01–7.91 (2H, m, H-6B, H-5A), 7.73 (1H, d, *J* = 0.8 Hz, H-3A), 6.75 (1H, d, *J* = 5.9 Hz, H-5B), 6.22 (1H, dt, *J* = 5.6, 2.0 Hz, H-2′), 6.08–6.06 (1H, m, H-3′), 5.40–5.38 (1H, m, H-1′), 4.81–4.80 (1H, m, H-4′), 3.03–2.93 (1H, m, H-5′a), 1.96–1.83 (1H, m, H-5′b). ^13^C NMR (75.5 MHz, CD_3_OD) δ: 153.3, 146.7, 145.6, 138.3, 137.9, 131.4, 127.1, 115.6, 113.7, 109.2, 74.3, 65.5, 41.0. HRMS *m/z*: calculated for С_13_Н_14_N_4_O [М+H]^+^ 243.1240; found [М+H]^+^ 243.1242.

1-(4′-Hydroxy-2′-cyclopenten-1′-yl)-4-(2-aminopyridin-3-yl)pyrazole **(9)**. White powder. Yield 63%. ^1^H NMR (300 MHz, CDCl_3_) δ: 8.01 (1H, dd, *J* = 5.0, 1.8 Hz, H-6B), 7.77–7.60 (2H, m, H-3A, H-5A), 7.39 (1H, dd, *J* = 7.4, 1.8 Hz, H-4B), 6.71 (1H, dd, *J* = 7.4, 5.0 Hz, H-5B), 6.34–6.31 (1H, m, H-2′), 5.99 (1H, dd, *J* = 5.5, 2.5 Hz, H-3′), 5.23–5.18 (1H, m, H-1′), 4.77 (1H, dt, *J* = 6.7, 2.0 Hz, H-4′), 4.65 (2H, s, NH_2_), 2.74–2.64 (1H, m, H-5′a), 2.11–2.04 (1H, m, H-5′b). ^13^C NMR (75.5 MHz, CDCl_3_) δ: 156.1, 146.7, 139.3, 138.6, 137.2, 131.7, 127.1, 118.3, 114.7, 74.6, 65.7, 40.6, 29.7. HRMS *m/z*: calculated for С_13_Н_14_N_4_O [М+H]^+^ 243.1240; found [М+H]^+^ 243.1246.

1-(4′-Hydroxy-2′-cyclopenten-1′-yl)-4-(4-aminopyrimidin-5-yl)pyrazole (**10**). White powder. Yield 80%. ^1^H NMR (300 MHz, CD_3_OD) δ: 8.36 (1H, s, H-2B), 8.13 (1H, s, H-5A), 7.99 (1H, s, H-6B), 7.77 (1H, s, H-3A), 6.24–6.21 (1H, m, H-2′), 6.09–6.06 (1H, m, H-3′), 5.41–5.38 (1H, m, H-1′), 4.86–4.82 (1H, m, H-4′), 2.98–2.93 (1H, m, H-5′a), 1.89 (1H, m, H-5′b). ^13^C NMR (75.5 MHz, CD_3_OD) δ: 161.7, 155.1, 151.0, 138.5, 137.8, 131.3, 127.4, 113.9, 110.8, 74.3, 65.6, 41.0. HRMS *m/z*: calculated for С_12_Н_13_N_5_O [М+H]^+^ 244.1193; found [М+H]^+^ 244.1192.

### General Procedure for the Synthesis of Compounds 1-3

Compounds **8–10** (0.2–0.3 mmol) were dissolved in dioxane: water (10:1). N-Methylmorpholine N-oxide (10 equiv.) and osmium tetroxide (0.25 equiv.) were added to the solution. The reaction mixture was stirred for 6 h and solvents were evaporated. The residues were dissolved in methanol: water (1:1) system (50 ml) and purified on Dowex 50^+^ eluted first with water and then with NH_3_ aq (2%) to give the products **1-3** with 72–85% yields.

1-(2′,3′,4′-Trihydroxycyclopent-1′-yl)-4-(4-aminopyridin-3-yl)pyrazole **(1)**. Colorless oil. Yield 72%. ^1^H NMR (300 MHz, CD_3_OD) δ: 8.08 (1H, s, H-2B), 8.01–7.97 (2H, m, H-6B, H-5A), 7.75 (1H, s, H-3A), 6.83 (1H, d, *J* = 6.2 Hz, H-5B), 4.68 (1H, dt, *J* = 9.4, 7.9 Hz, H-2′), 4.48 (1H, dd, *J* = 7.7, 5.0 Hz, H-3′), 4.12–4.08 (1H, m, H-1′), 3.97–3.95 (1H, m, H-4′), 2.81–2.71 (1H, m, H-5′a), 2.09–2.02 (1H, m, H-5b). ^13^C NMR (75.5 MHz, CD_3_OD) δ: 155.2, 143.8, 142.9, 138.2, 128.7, 114.2, 114.0, 109.1, 77.1, 76.4, 74.1, 65.9, 36.0. HRMS *m/z*: calculated for С_13_Н_16_N_4_O_3_ [М+H]^+^ 277.1295; found [М+H]^+^ 277.1298.

1-(2′,3′,4′-Trihydroxycyclopent-1′-yl)-4-(2-aminopyridin-3-yl)pyrazole **(2)**. Pale yellow oil. Yield 77%.^1^H NMR (300 MHz, CD_3_OD) δ: 8.01 (1H, s, H-5A), 7.91–7.87 (1H, m, H-6B), 7.78 (1H, s, H-3A), 7.59 (1H, dd, *J* = 7.4, 1.8 Hz, H-4B), 6.76 (1H, dd, *J* = 7.4, 5.2 Hz, H-5B), 4.68–4.65 (1H, m, H-2′), 4.47 (1H, dd, *J* = 7.6, 5.0 Hz, H-3’), 4.20–4.03 (1H, m, H-1′), 3.96–3.95 (1H, m, H-4′), 2.76–2.73 (1H, m, H-5′a), 2.13–1.97 (1H, m, H-5′b). ^13^C NMR (75.5 MHz, CD_3_OD) δ: 155.6, 143.4, 137.5, 137.4, 127.7, 116.9, 113.8, 113.1, 76.6, 75.8, 73.6, 68.8, 35.5. HRMS *m/z*: calculated for С_13_Н_16_N_4_O_3_ [М+H]^+^ 277.1295; found [М+H]^+^ 277.1293.

1-(2′,3′,4′-Trihydroxycyclopent-1′-yl)-4-(4-aminopyrimidin-3-yl)pyrazole **(3)**. Pale yellow oil. Yield 85%. ^1^H NMR (300 MHz, CD_3_OD) δ: 8.34 (1H, s, H-2B), 8.13 (1H, s,H-5A), 8.02 (1H, s, H-6B), 7.78 (1H, s, H-3A), 4.67 (1H, dt, *J* = 9.3, 7.8 Hz, H-2′), 4.47 (1H, dd, *J* = 7.7, 5.0 Hz, H-3′), 4.12–4.08 (1H, m, H-1′), 3.97–3.95 (1H, m, H-4′), 2.81–2.70 (1H, m, H-5′a), 2.08–1.99 (1H, m, H-5′b). ^13^C NMR (75.5 MHz, CD_3_OD) δ: 161.6, 155.6, 151.9, 138.0, 128.6, 113.7, 110.9, 77.1, 76.3, 74.1, 65.9, 36.0. HRMS *m/z*: calculated for С_12_Н_15_N_5_O_3_ [М+H]^+^ 278.1248; found [М+H]^+^ 278.1268.

### Enzyme Inhibition

Each reaction mixture (100 μl, 50 mM KH_2_PO_4_, pH 7.0) contained *E. coli* purine nucleoside phosphorylase (0.00056 μg), 0.2 mM inosine and 10 mM of tested compounds **1–3**, **8–10**. For kinetic parameters: each reaction mixture (50 μl, 50 mM KH_2_PO_4_, pH 7.0) contained *E. coli* purine nucleoside phosphorylase (0.00028 μg), inosine (0.005–2 mM) and inhibitor (0, 2, 4 or 10 mM of **1**, **2** or **3**). Specific activity of the PNP is 50 μmol/min*mg. Reaction mixtures were incubated 2 min at 37 C. Substrate and product quantities were determined using HPLC (Waters 1,525, column Ascentis Express C18, 2.7 μm, 3.0 × 75 mm, eluent A 0.1% aqeous TFA, eluent B 0.1% TFA/70% acetonitrile in water, detection at 254 nm, UV-detector Waters 2,489). Each experiment was repeated three times. Kinetic parameters were determined by nonlinear regression analysis using SciDAVis v2.3.0 software. Simple equation for non-competitive inhibition was used. V = Vmax*S/[(KM + S)*(1 + Ci/Ki)]. Catalytic constants (k_cat_) were calculated per 1 subunit (26 kDa, calculated based on amino acid sequence).

## Results and Discussion

### Chemistry

**SCHEME 1:**
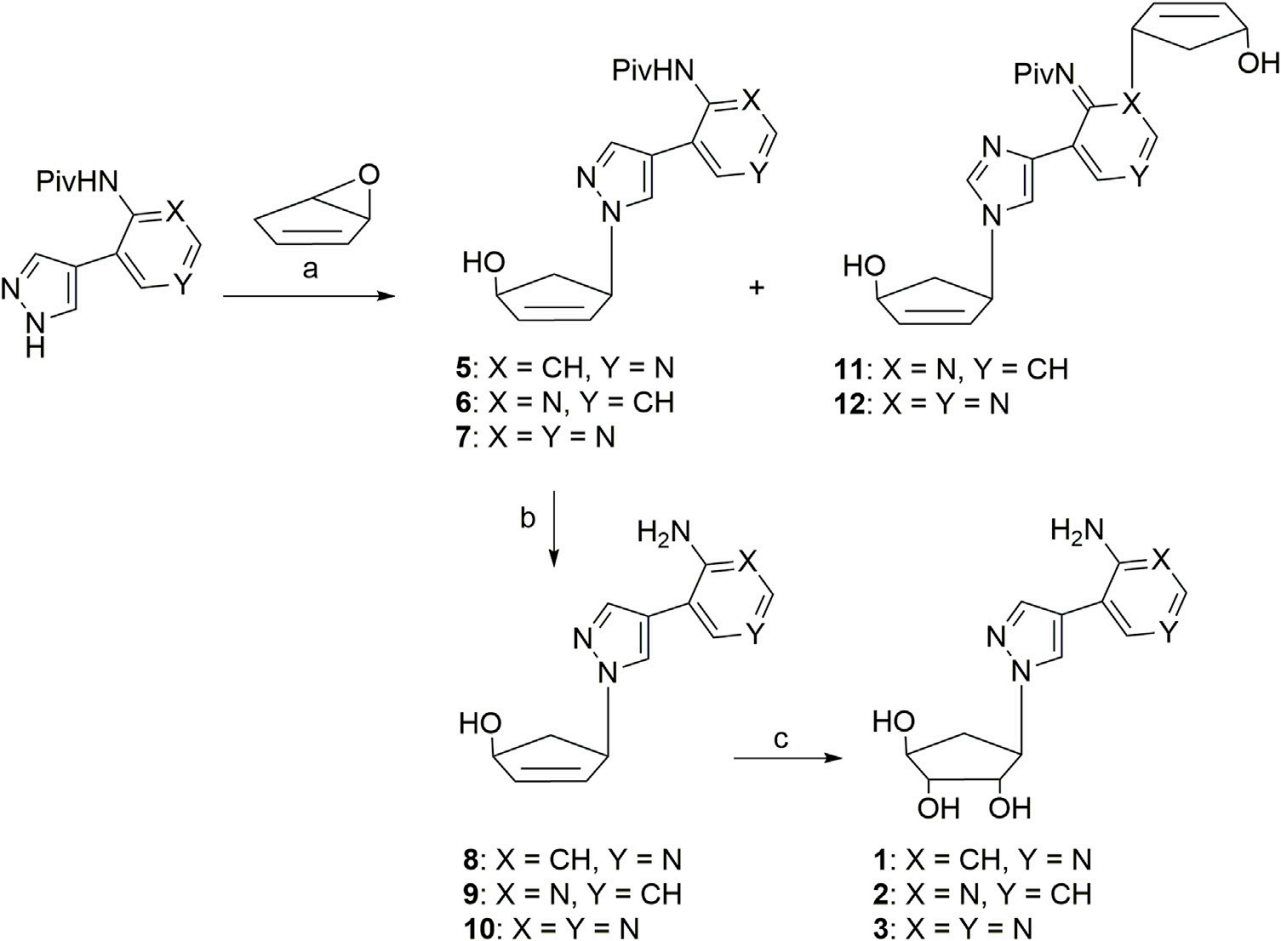
Reagents and conditions: a) Pd(PPh_3_)_4_, DMF/THF; b) K_2_CO_3_, CH_3_OH, reflux; c) OsO_4_, dioxane/H_2_O.

Target compounds **1-3** were synthesized from the known precursor of many 5′-norcarbocyclic nucleosides, 6-oxybicyclo[3.1.0.]hex-2-ene (Scheme 1) (Korach et al., 1973). Condensation with various pyrazole-containing fleximer bases (Khandazhinskaya et al., 2021) was carried out under Trost reaction conditions (Trost et al., 1988).

The pivaloyl residue on the amino group of compounds **5–7** was removed by reflux with K_2_CO_3_ in methanol to give intermediates **8–10**. Finally, to obtain nucleoside analogs 1–3, compounds **8–10** were treated with osmium tetroxide in dioxane:water (10:1). The oxidized products were then isolated on Dowex H+ with a 72–85% yield.

Typically when performing the Trost reaction a 1.5-fold excess of 6-hydroxybicyclo[3.1.0.]hex-2-ene is used. Thus, for the synthesis of compounds **6-7,** in addition to the mono-substituted products of the 5′-norcarbocyclic residue, it was not surprising that double addition to the hydroxycyclopentenyl group occurred, producing compounds **11–12** (Scheme 1). One carbocyclic residue was attached to the pyrazole ring and a second to either the N1 or N3 nitrogen atom of the pyridine/pyrimidine ring. The ^1^H NMR spectra of compounds **11–12** revealed a doubling of the carbocyclic residue signals.

It was worthwhile to note that in the case of compound **5**, where the CH in the pyrimidine ring is located near the substituted amino group, the formation of bis-substituted products was not observed. The structures of all new compounds were confirmed with ^1^H NMR, ^13^C NMR and HRMS.

### Inhibition Studies

**FIGURE 2:**
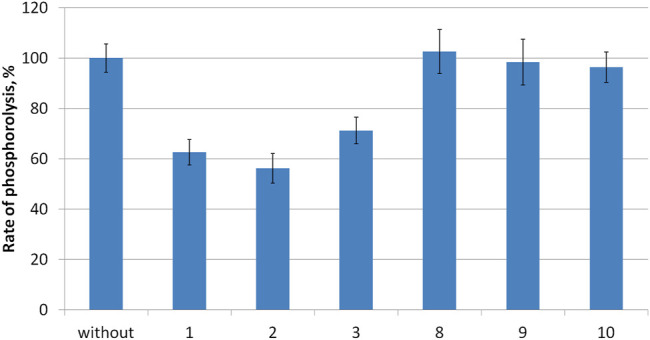
Rate of inosine phosphorolysis and kinetic parameters of inosine phosphorolysis inhibition.

**FIGURE 3:**
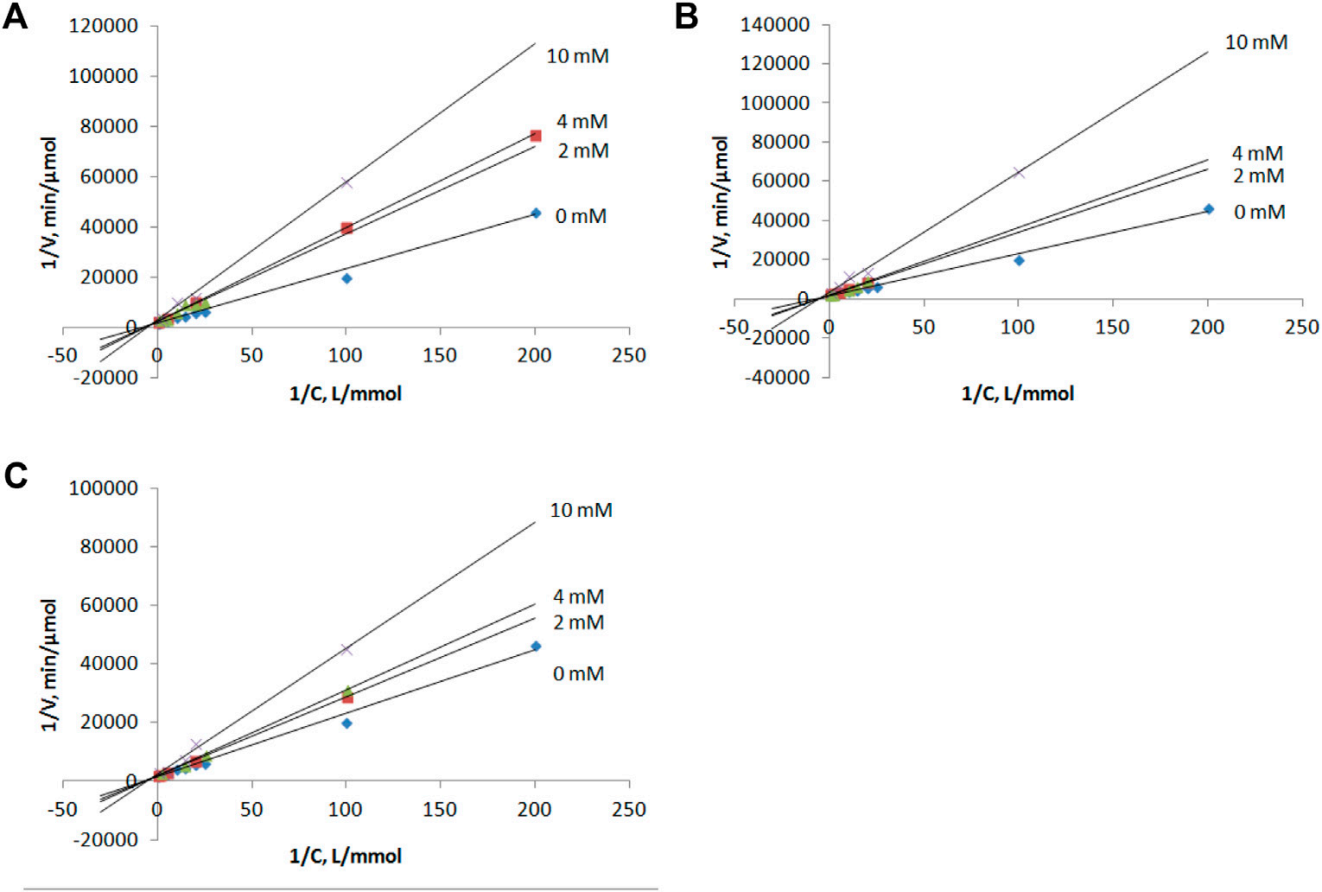
Lineweaver–Burk plots for the inosine phosphorolysis at concentrations of compounds **1**, **2** and **3** equal to 0, 2, 4 and 10 mM. **(A)** compound **1**, **(B)** compound **2**, **(C)** compound **3**.

Compounds **1–3** as well as intermediates **8–10** were then studied as potential inhibitors of PNP *E. coli* (Figure 2). Analog **1-3** were found to be weak, non-competitive inhibitors with inhibition constants of 24 ± 3 **(3)**, 17 ± 4 **(1)**, and 14 ± 1 **2**) mM (Table 1; Figure 3). Compounds **8–10** lack any inhibitory effect under the experimental conditions. PNP *E. coli* is high stable in tested conditions (Lee, 2001).

**TABLE 1:** Kinetic parameters of inosine phosphorolysis inhibition.

Inhibitor	Inhibitor concentration, mM	K_M_, mM	k_cat_, s^−1^	K_i_, mM
No comp.	-	0.13 ± 0.01	900 ± 60	-
**1**	2	0.16 ± 0.01	830 ± 50	17 ± 4
	4	0.14 ± 0.03	680 ± 160	
	10	0.16 ± 0.04	560 ± 140	
**2**	2	0.15 ± 0.02	800 ± 110	14 ± 1
	4	0.15 ± 0.02	720 ± 70	
	10	0.16 ± 0.03	500 ± 80	
**3**	2	0.12 ± 0.01	840 ± 70	24 ± 3
	4	0.13 ± 0.01	750 ± 50	
	10	0.13 ± 0.02	660 ± 110	

Each reaction mixture (100 μL, 50 mM KH_2_PO_4_, pH 7.0) contained *E. coli* purine nucleoside phosphorylase (0.00056 μg), 0.2 mM inosine and 10 mM of tested compounds **1–3**, **8–10**. Reaction mixtures were incubated 2 min at 37 C.

Each reaction mixture (50 μl, 50 mM KH_2_PO_4_, pH 7.0) contained *E. coli* purine nucleoside phosphorylase (0.00028 μg), inosine (0.005–2 mM) and inhibitor (0, 2, 4 or 10 mM of **1**, **2** or **3**). Reaction mixtures were incubated 2 min at 37 C.

**FIGURE 4:**
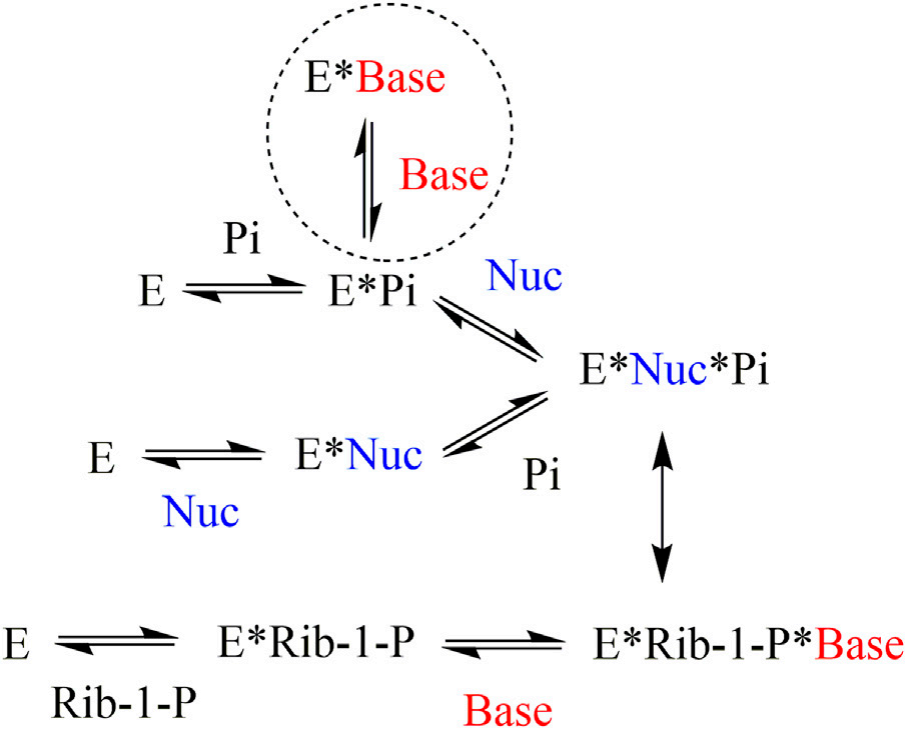
Mechanism of substrate binding in the active site of purine nucleoside phosphorylase from bacterial sources (Jensen, 1976). E–enzyme, Base–heterocyclic base, Nuc–nucleoside, Rib-1-P–*α*- *d* -ribose-1-phosphate, Pi–inorganic phosphate.

In the case of PNP, non-competitive inhibition of nucleoside phosphorolysis was observed for the heterocyclic bases. It should be noted that non-competitive inhibition of nucleoside phosphorolysis observed in the experiment is not a true non-competitive mechanism. This can be explained by the order of substrate binding, which differed in the reactions of phosphorolysis and nucleoside synthesis (Figure 4) (Jensen, 1976). If, during phosphorolysis reaction, a random order of substrate binding is observed (both the nucleoside and inorganic phosphate can bind first), then in the process of nucleoside synthesis, ribose-1-phosphate is always bound first, followed by the heterocyclic base. The heterocyclic base needs a phosphate group to bind to the PNP active site, either in the form of an inorganic phosphate or as part of ribose-1-phosphate. Thus, if inorganic phosphate is added first during nucleoside phosphorolysis, then the heterocyclic base and nucleoside compete for binding to the active site. If the nucleoside is added first during nucleoside phosphorolysis, then the heterocyclic base is unable to inhibit this process.

When studying the effect of a particular heterocyclic base on nucleoside phosphorolysis, the same case is observed as is seen with non-competitive inhibition that is, the reaction rate decreases, and the Michaelis constant does not change. These data suggest that compounds **1–3** interact with the active site of the enzyme like natural heterocyclic bases, i.e., only in the presence of a phosphate group in the active site. In the experiment, the concentration of phosphate relative to the inhibitor was in a fivefold excess. A similar situation was previously described for 7-deazahypoxanthine, which is also a non-competitive inhibitor of this enzyme with *Ki* 0.13 mM (Timofeev et al., 2018).

From the data obtained, it can be assumed that the new 5′-norcarbocyclic nucleoside analogs interact with the active site of the *E. coli* PNP similarly to the normal heterocyclic bases. In addition, fleximer heterocyclic bases without a carbocyclic moiety exhibited substrate properties with respect to PNP as well. Interestingly, despite their flexibility, the fleximers do indeed exhibit selectivity and do not randomly bind with every enzyme. Moreover, in some cases it has been shown that there is a strong synergism between the fleximer and the enzyme binding, with the binding site dictating the conformation of the fleximer, as well as to lead to unexpected inhibition (Seley et al., 2003). An example of this was seen by Seley-Radtke and Plavek (Polak et al., 2004) where the guanosine fleximer adopted an unusual syn conformation in S-adenosylhomocysteine hydrolase, but strongly preferred an anti conformation in solution, thus indicating the enzyme was exhibiting an influence on the conformation of the fleximer. Although we do not yet have a crystal or NMR structure of our compounds in PNP, once we have that available, it will be interesting to see if that same trend holds for our compounds and PNP.

The addition of compounds **1**-**3** to the reaction mixture in the presence of human PNP did not lead to a decrease in its rate. Mammalian PNP’s has limited substrate specificity compared to bacterial PNP’s. It is likely that these compounds are unable to bind to the active site of the human protein.

It should be noted that the presence of a cyclopentyl moiety with 2′ and 3′ hydroxyls is necessary for the inhibitory properties, since compounds **8–10**, without those groups did not exhibit an inhibitory effect under the experimental conditions. On the other hand we didn’t observe the product formation in reaction mixtures with compounds **8–10** and *E. coli* PNP. This correlated with literature data, for example, the carbocyclic analogue of 2′,3′-dideoxy-2′,3′-didehydroguanosine (carbovir, a nucleoside inhibitor of HIV reverse transcriptase) also does not exhibit inhibitory properties against human PNP (Marr and Penn, 1992) while many other analog of guanosine possessing the hydroxyl groups have proven to be very effective PNP inhibitors (Morris and Montgomery, 1998).

## Conclusion

Thus, several new fleximer 5′-norcarbocyclic aza/deazapurine nucleoside analogs were synthesized. The fleximer nucleosides proved to be non-competitive inhibitors of *E. coli* PNP, which bound in the active site similar to natural heterocyclic bases, although the fleximer aza/deazapurine bases themselves served as substrates of the enzyme. It was also shown that the two hydroxyl groups attached to the 5′-norcarbocyclic fragment is necessary for the inhibitory properties of these novel compounds. Replacing of the 2′-deoxyribo- or ribofuranose by 5′-norcarbocyclic moiety lead to some inhibitory properties and open the direction for future investigation.

## Data Availability

The original contributions presented in the study are included in the article/Supplementary Materials, further inquiries can be directed to the corresponding author.
