# Supplementary material for: Synthesis of New 5′-Norcarbocyclic Aza/Deaza Purine Fleximers - Noncompetitive Inhibitors of E.coli Purine Nucleoside Phosphorylase
Source: Front Chem. 2022 May 4;10:867587. doi: 10.3389/fchem.2022.867587 (PMC9114674; doi:10.3389/fchem.2022.867587)

Supplementary Material

Synthesis of new flexible 5'-norcarbocyclic aza/dezaza-purine nucleoside analogues - noncompetitive inhibitors of *E.coli* purine nucleoside phosphorylase

Anastasia Khandazhinskaya[1], Ilja Fateev[2], Irina Konstantinova[2], Roman S. Esipov[2], Konstantin Polyakov[1], Katherine Seley-Radtke[3], Sergey Kochetkov[1], Elena Matyugina[1]

[1]Engelhardt Institute of Molecular Biology of the Russian Academy of Sciences, 32 Vavilov St., Moscow 119991, Russia
[2]Shemyakin-Ovchinnikov Institute of Bioorganic Chemistry, Russian Academy of Sciences, Moscow 117997, Russia
[3]Department of Chemistry & Biochemistry, University of Maryland, Baltimore County, 1000 Hilltop Circle, Baltimore, MD 21250, USA

Table of Content

Figure S1 S2
General information S3
Characterization data of products **5-7, 11,12** S3
^{1}H-NMR spectrum (300 MHz, $CDCl_3$) of compound **5** S6
^{13}C-NMR spectrum (75.5 MHz, $CDCl_3$) of compound **5** S6
^{1}H-NMR spectrum (300 MHz, $CDCl_3$) of compound **6** S7
^{13}C-NMR spectrum (75.5 MHz, $CDCl_3$) of compound **6** S7
^{1}H-NMR spectrum (300 MHz, $CDCl_3$) of compound **7** S8
^{13}C-NMR spectrum (75.5 MHz, $CDCl_3$) of compound **7** S8
^{1}H-NMR spectrum (300 MHz, $CDCl_3$) of compound **8** S9
^{13}C-NMR spectrum (75.5 MHz, $CDCl_3$) of compound **8** S9
^{1}H-NMR spectrum (300 MHz, $CDCl_3$) of compound **9** S10
^{13}C-NMR spectrum (75.5 MHz, $CDCl_3$) of compound **9** S10
^{1}H-NMR spectrum (300 MHz, CD_3OD) of compound **10** S11
^{13}C-NMR spectrum (75.5 MHz, CD_3OD) of compound **10** S11
^{1}H-NMR spectrum (300 MHz, CD_3OD) of compound **1** S12
^{13}C-NMR spectrum (75.5 MHz, CD_3OD) of compound **1** S12
^{1}H-NMR spectrum (300 MHz, CD_3OD) of compound **2** S13
^{13}C-NMR spectrum (75.5 MHz, CD_3OD) of compound **2** S13
^{1}H-NMR spectrum (300 MHz, CD_3OD) of compound **3** S14
^{13}C-NMR spectrum (75.5 MHz, CD_3OD) of compound **3** S14

Figure S1. Structures of some PNP inhibitors

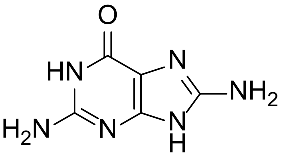

IC_{50}= 0.2 μM

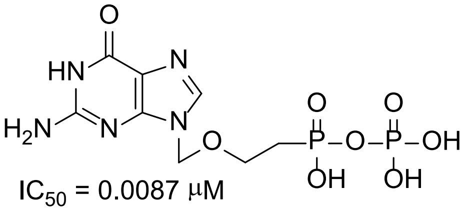

IC_{50} = 0.0087 μM

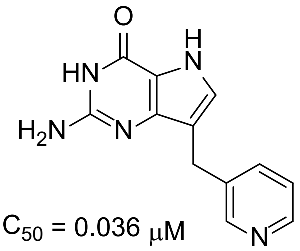

IC_{50} = 0.036 μM

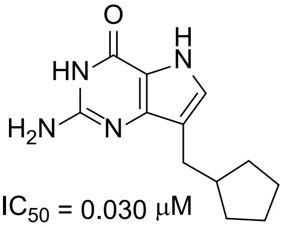

IC_{50} = 0.030 μM

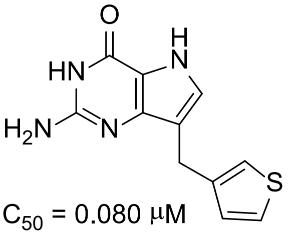

IC_{50} = 0.080 μM

General information

The reactions were performed with commercial reagents (Acros, Aldrich, and Fluka); anhydrous solvents were purified according to the standard procedures. Column chromatography was performed on Silica Gel 60 0.040–0.063 mm (Merck, Germany) columns, Dowex-50 (H^+). Preparative liquid chromatography (PLC) was performed on Silica Gel 60 F_{254} with concentrating zone glass plates (Merck, Germany). Thin layer chromatography (TLC) was performed on Silica Gel 60 F_{254} aluminium-backed plates (Merck, Germany).

NMR spectra were recorded on Bruker Avance III spectrometer (Bruker BioSpin, Rheinstetten, Germany) with an operating frequency of 300 MHz for 1H-NMR and 75.5 MHz for 13C-NMR in $CDCl_3$, CD_3OD or DMSO-d_6.

High resolution mass spectra (HRMS) were obtained on a Bruker Daltonics micrOTOF-Q II instrument using electrospray ionization (ESI). The measurements were acquired in a negative ion mode with the following parameters: interface capillary voltage – 3700 V; mass range from *m/z* 50 to 3000; external calibration (Electrospray Calibrant Solution, Fluka); nebulizer pressure – 0.3 Bar; flow rate – 3 µL/min; dry gas nitrogen (4.0 L/min); interface temperature was set at 180 or 190 ºC. A syringe injection was used.

Characterization data of products 5-7, 11, 12

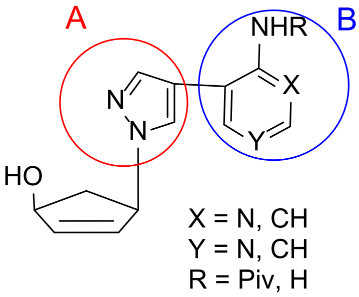

1-(4’-Hydroxy-2’-cyclopenten-1’-yl)-4-(4-pivaloylaminopyridin-3-yl)pyrazole (5). Compound **5** was synthesized from 4-(4-pivaloylaminopyridin-3-yl)-1*H*-pyrazole (120 mg, 0.49 mmol) and 6-oxybicyclo[3.1.0.]hex-2-ene (60 mg, 0.74 mmol). Purification on the silica gel column eluting with chloroform: methanol (95:5) gave **5** as a pale, yellow powder (110 mg, 68%).

^{1}H NMR (300 MHz, $CDCl_3$) δ: 8.44 (1H, d, *J* = 5.9 Hz, H-2B), 8.39-8.37 (2H, m, H-6B, H-5A), 7.84 (1H, s, NH-B), 7.67 (1H, s, H-3A), 7.63 (1H, s, H-5B), 6.33 (1H, dt, *J* = 5.5, 1.9 Hz, H-2’), 5.99 (1H, dd, *J* = 5.5, 2.5 Hz, H-3’), 5.31 – 5.26 (1H, m, H-1’), 4.83 – 4.79 (1H, m, H-4’), 4.21 (1H, brs, OH), 2.81-2.72 (1H, m, H-5’a), 2.09-2.03 (1H, m, H-5’b), 1.20 (s, 9H, Piv). ^{13}C NMR

(75.5 MHz, $CDCl_3$) δ: 176.5, 149.5, 149.1, 142.5, 139.1, 138.2, 131.0, 127.4, 117.0, 113.8, 113.1, 74.1, 65.5, 40.2, 39.7, 26.8.

1-(4'-Hydroxy-2'-cyclopenten-1'-yl)-4-(2-pivaloylaminopyridin-3-yl)pyrazole (6). Compound **6** was synthesized from 4-(2-pivaloylaminopyridin-3-yl)-1*H*-pyrazole (90 mg, 0.36 mmol) and 6-oxybicyclo[3.1.0.]hex-2-ene (44 mg, 0.54 mmol). Purification on the silica gel column eluting with chloroform: methanol (95:5) gave **6** as a white powder (90 mg, 75%).

^{1}H NMR (300 MHz, $CDCl_3$) δ: 8.40 (1H, dd, J = 5.0, 1.8 Hz, H-6B), 7.74 – 7.66 (2H, m, H-4B, H-3A), 7.64 (1H, s, H-5A), 7.20 (1H, dd, J = 7.7, 4.8 Hz, H-5B), 6.36 – 6.26 (1H, m, H-2'), 5.97 (1H, dd, J = 5.5, 2.5 Hz, H-3'), 5.23-5.18 (1H, m, H-1'), 4.77 (1H, dt, J = 6.9, 2.0 Hz, H-4'), 2.71 (1H, m, H-5'a), 2.12 – 1.98 (1H, m, H-5'b), 1.26 (9H, s, Piv). ^{13}C NMR (75.5 MHz, $CDCl_3$) δ: 176.2, 147.7, 146.1, 138.8, 138.1, 137.9, 131.1, 127.0, 123.3, 120.9, 117.3, 74.1, 65.2, 40.1, 39.1, 26.9.

1-(4'-Hydroxy-2'-cyclopenten-1'-yl)-4-(4-pivaloylaminopyrimidin-5-yl)pyrazole (7). Compound **7** was synthesized from 4-(4-pivaloylaminopyrimidin-5-yl)-1*H*-pyrazole (100 mg, 0.41 mmol) and 6-oxybicyclo[3.1.0.]hex-2-ene (50 mg, 0.6 mmol). After purification on the silica gel column eluting with chloroform: methanol (95:5), **7** was obtained as a white powder (107 mg, 79%).

^{1}H NMR (300 MHz, $CDCl_3$) δ: 8.97 (1H, s, H-2B), 8.60 (1H, s, H-5A), 8.00 (1H, s, NH), 7.77 (1H, s, H-6B), 7.67 (1H, s, H-3A), 6.34 (1H, dt, J = 5.5, 1.9 Hz, H-2'), 6.00 (1H, dd, J = 5.5, 2.5 Hz, H-3'), 5.31-5.26 (1H, m, H-1'), 4.88-4.84 (1H, m, H-4'), 2.82-2.72 (1H, m, H-5'a), 2.08 (1H, d, J = 14.6 Hz, H-5'b), 1.26 (9H s, Piv). ^{13}C NMR (75.5 MHz, $CDCl_3$) δ: 156.6, 155.5, 139.6, 138.1, 131.5, 127.6, 117.5, 113.8, 74.6, 66.1, 40.6, 40.4, 27.3.

1-(4'-Hydroxy-2'-cyclopenten-1'-yl)-4-(1-(4''-Hydroxy-2'-cyclopenten-1''-yl)-2-(pivaloylamino) pyridin-3-yl)pyrazole (11). Compound **11** was synthesized as byproduct in reaction of 4-(2-pivaloylaminopyridin-3-yl)-1*H*-pyrazole (90 mg, 0.36 mmol) and 6-oxybicyclo[3.1.0.]hex-2-ene (44 mg, 0.54 mmol). Purification on the silica gel column eluting with chloroform: methanol (98:2) gave **12** as an off-white powder (31 mg, 21%).

^{1}H NMR (300 MHz, $CDCl_3$) δ: 8.36 (1H, dd, J = 4.8, 1.8 Hz, H-6B), 7.97 (1H, dd, J = 7.9, 1.8 Hz, H-4B), 7.85 (1H, s, H-3A), 7.76 (1H, d, J = 2.8 Hz, H-5A), 7.36 (1H, dd, J = 7.9, 4.7 Hz, H-5B), 6.36 – 6.34 (1H, m, H-2'), 5.98 (1H, dd, J = 5.5, 2.5 Hz, H-2') 5.95 – 5.82 (1H, m, H-3'), 5.42 – 5.10 (3H, m, H-3', 2xH-1'), 4.80 (1H, dt, J = 7.0, 1.8 Hz, H-4'), 4.55 (1H, dt, J = 6.2, 2.8 Hz, H-4'), 2.86 – 2.60 (2H, m, 2xH-5'a), 2.15 – 1.95 (2H, m, 2xH-5'b), 0.88 (9H, s, Piv). ^{13}C NMR (75.5 MHz, $CDCl_3$) δ: 146.0, 139.6, 139.5, 138.4, 136.1, 131.7, 131.5, 129.9, 129.8, 127.3, 124.4, 74.6, 66.0, 62.4, 62.3, 42.2, 42.1, 40.7, 40.6, 28.7, 28.3.

1-(4'-Hydroxy-2'-cyclopenten-1'-yl)-4-(1-(4''-Hydroxy-2'-cyclopenten-1''-yl)-4-(pivaloylamino)pyridin-5-yl)pyrazole (12). Compound **12** was synthesized as byproduct in reaction of 4-(4-pivaloylaminopyrimidin-5-yl)-1*H*-pyrazole (100 mg, 0.41 mmol) and 6-oxybicyclo[3.1.0.]hex-2-ene (50 mg, 0.6 mmol). After purification on the silica gel column eluting with chloroform: methanol (95:5), **11** was obtained as a pale yellow powder (18 mg, 14%).

^{1}H NMR (300 MHz, $CDCl_3$) δ: 8.96 (2H, d, *J* = 5.9 Hz, H-2B, H-5A), 7.9 (2H, d, *J* = 3.9 Hz, H-6B, H-3A), 6.32 (1H, dt, *J* = 5.5, 1.9 Hz, H-2'), 6.05 – 5.89 (2H, br s, H-2', H-3'), 5.41 (1H, s, H-3'), 5.36 – 5.20 (1H, m, H-1'), 5.0 (1H, s, H-1'), 4.81 (1H,dt, *J* = 6.9, 2.0 Hz, H-4'), 3.82 (2H, s, 2xOH), 2.82-2.71 (1H, m, H-5'a), 2.65-2.59 (1H, br s, H-5'a), 2.06-1.97 (2H, m, 2xH-5'b), 0.95 (9H s, Piv). ^{13}C NMR (75.5 MHz, $CDCl_3$) δ: 157.1, 156.0, 139.7, 139.6, 138.9, 138.0, 131.5 (2C), 130.0, 127.6 (2C), 114.0, 75.0, 74.6, 66.2, 63.8, 42.1, 40.7, 29.7, 28.6.

^{1}H-NMR spectrum (300 MHz, $CDCl_3$) of compound **5**

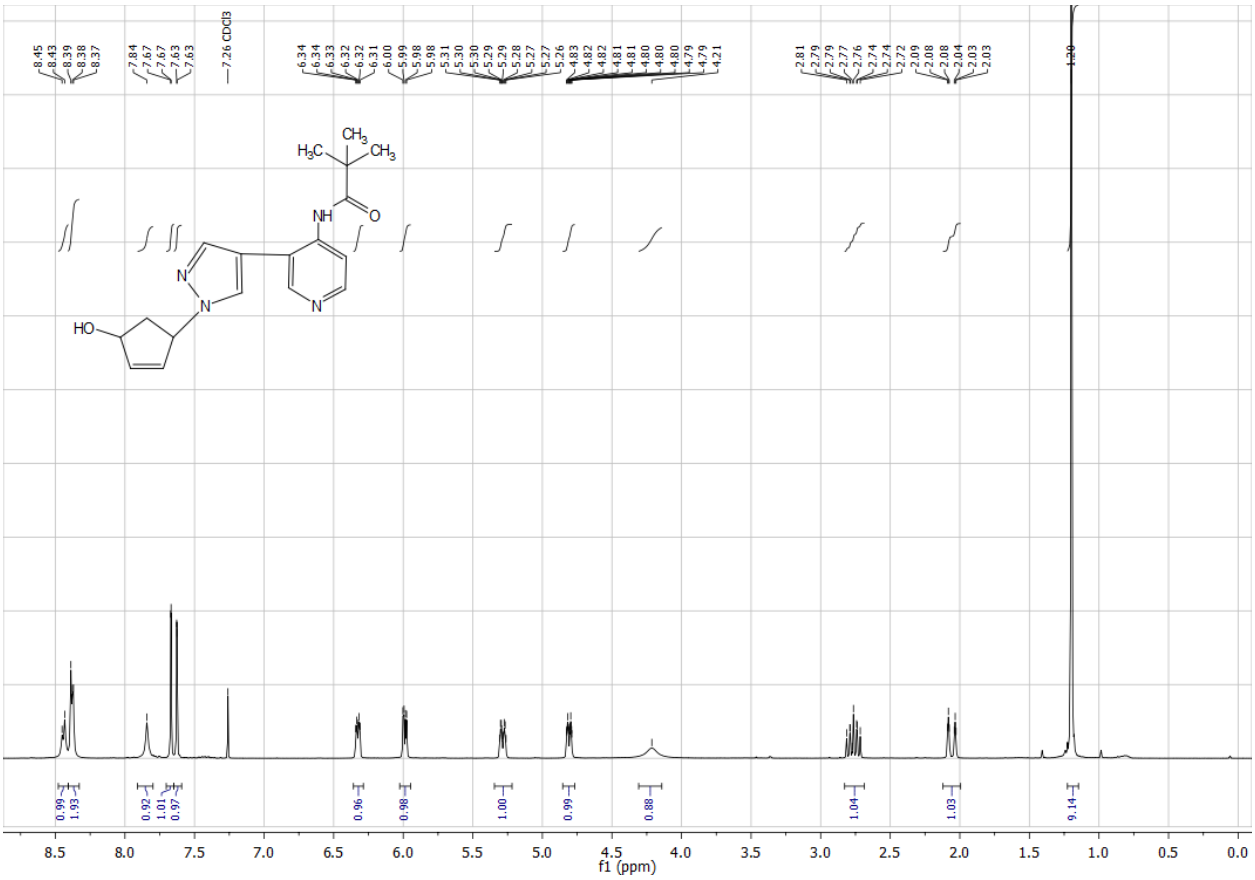

^{13}C-NMR spectrum (75.5 MHz, $CDCl_3$) of compound **5**

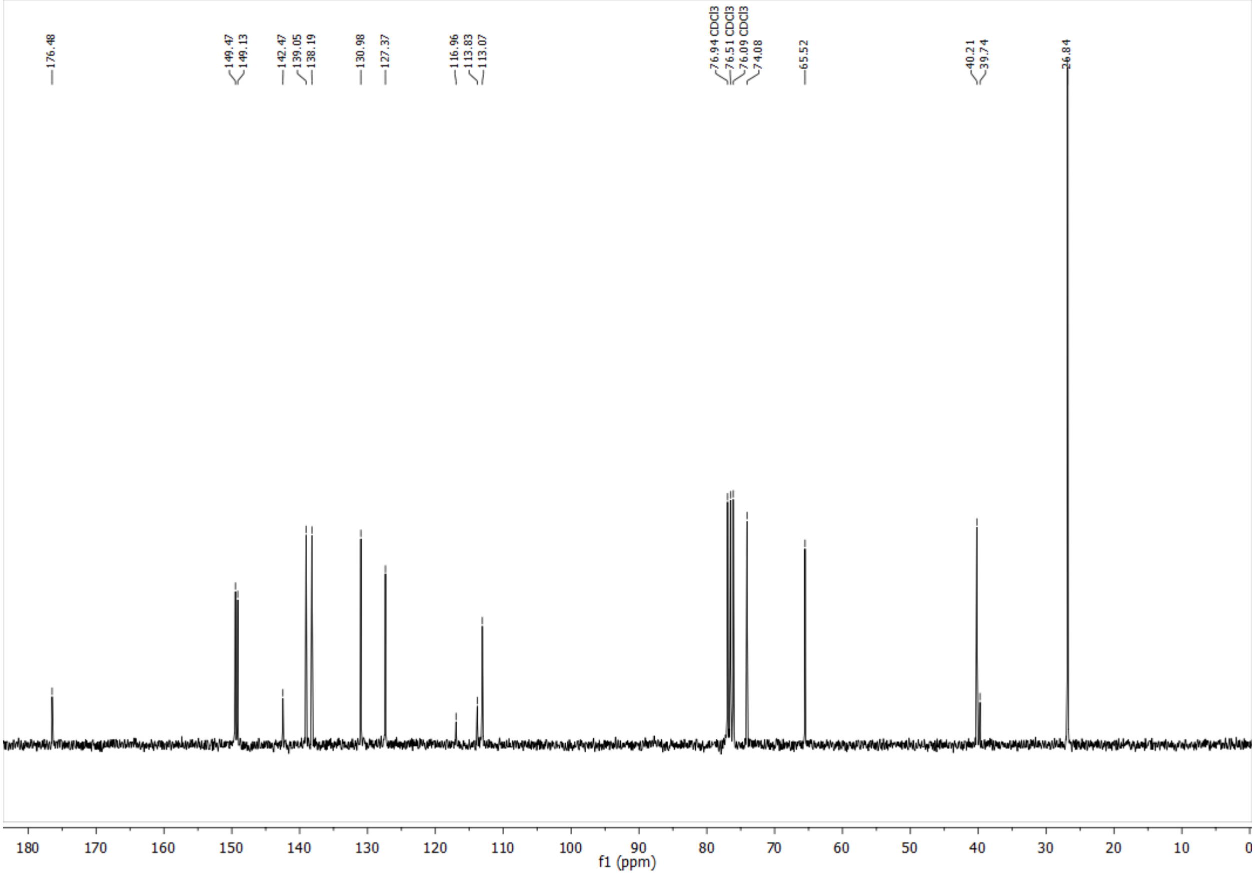

^{1}H-NMR spectrum (300 MHz, $CDCl_3$) of compound **6**

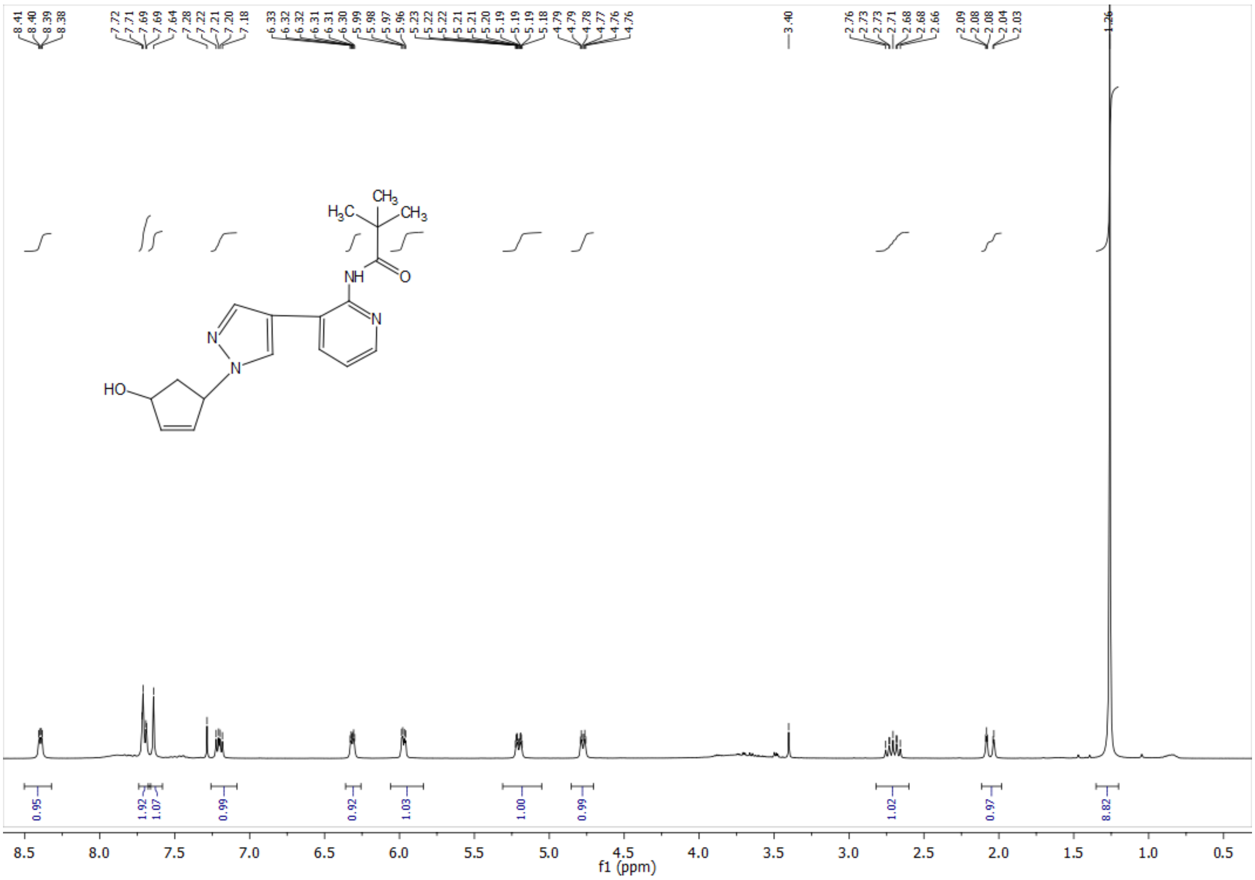

^{13}C-NMR spectrum (75.5 MHz, $CDCl_3$) of compound **6**

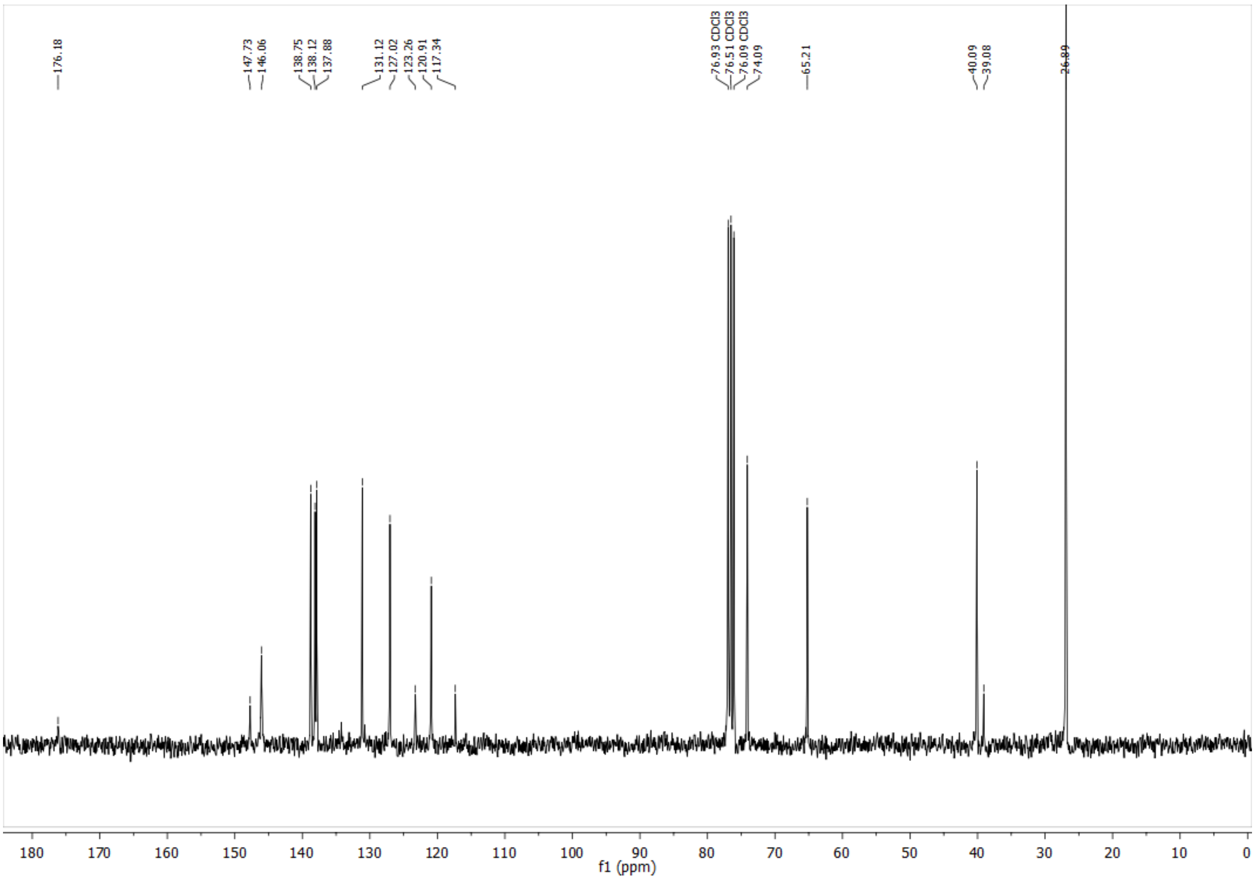

^{1}H-NMR spectrum (300 MHz, $CDCl_3$) of compound **7**

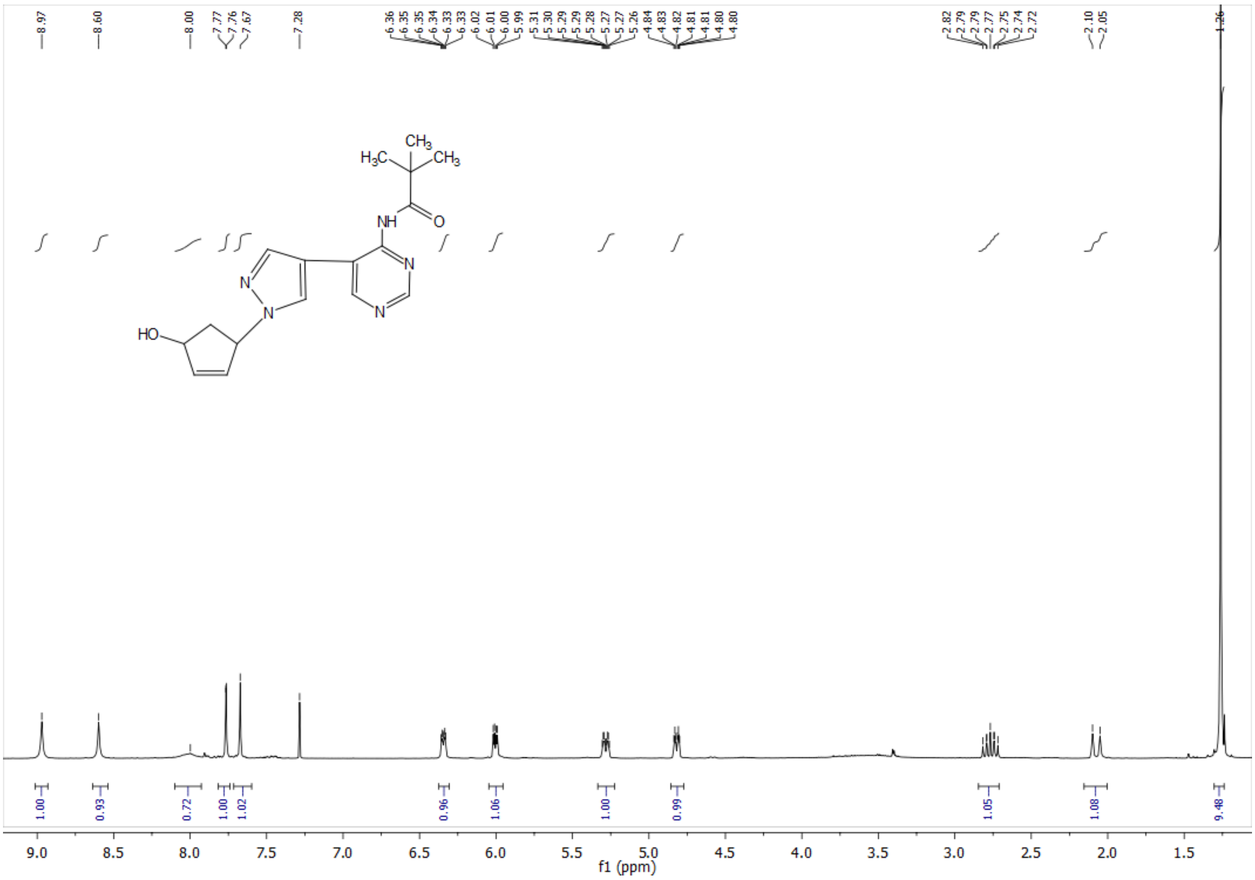

^{13}C-NMR spectrum (75.5 MHz, $CDCl_3$) of compound **7**

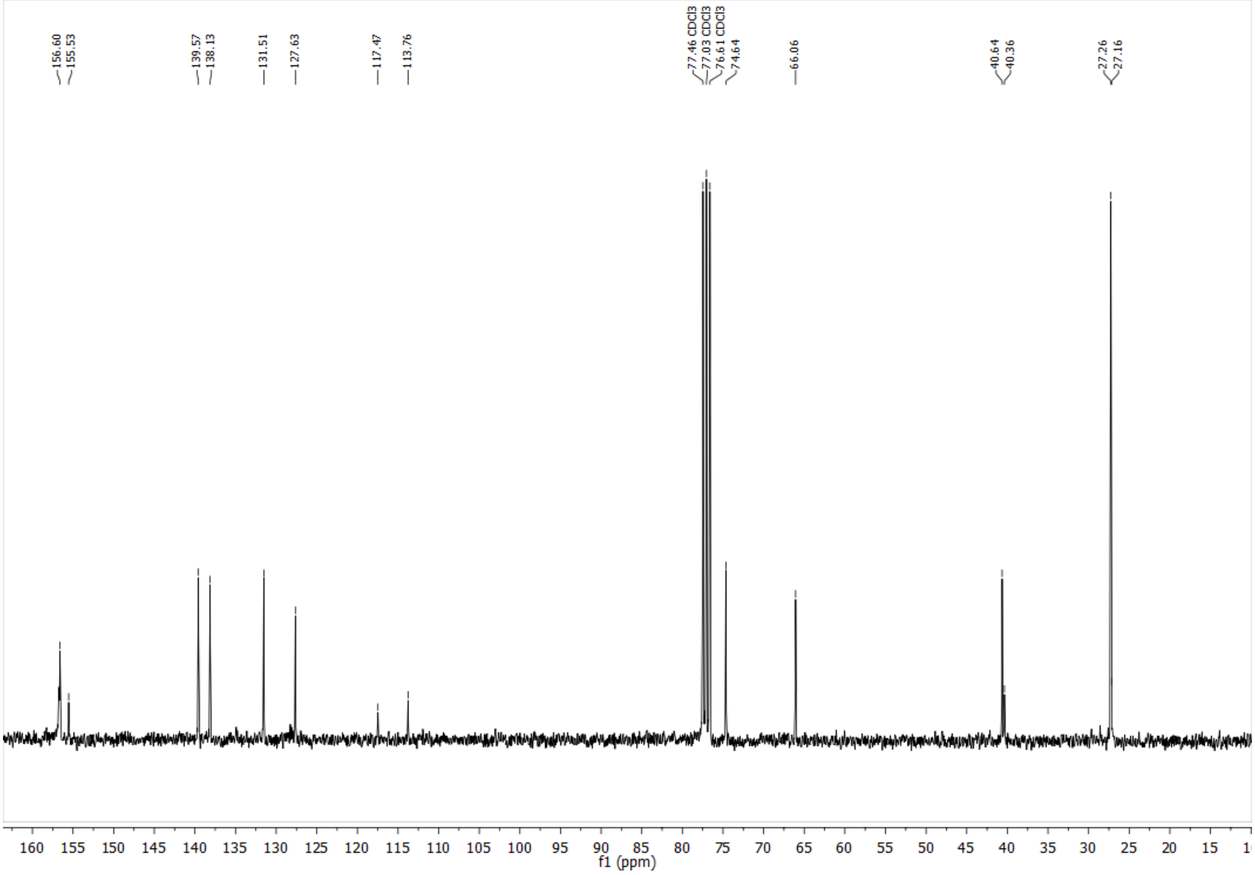

^{1}H-NMR spectrum (300 MHz, $CDCl_3$) of compound **8**

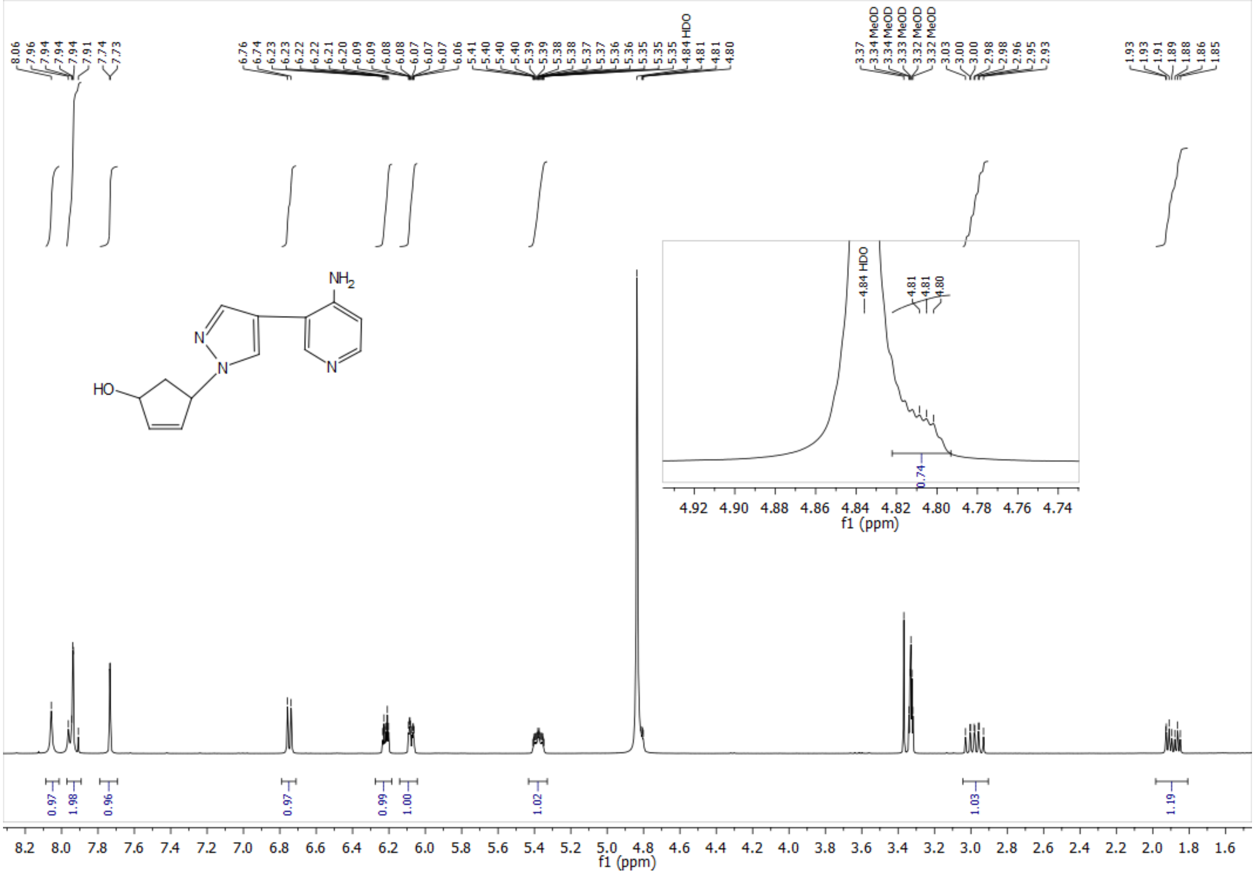

^{13}C-NMR spectrum (75.5 MHz, $CDCl_3$) of compound **8**

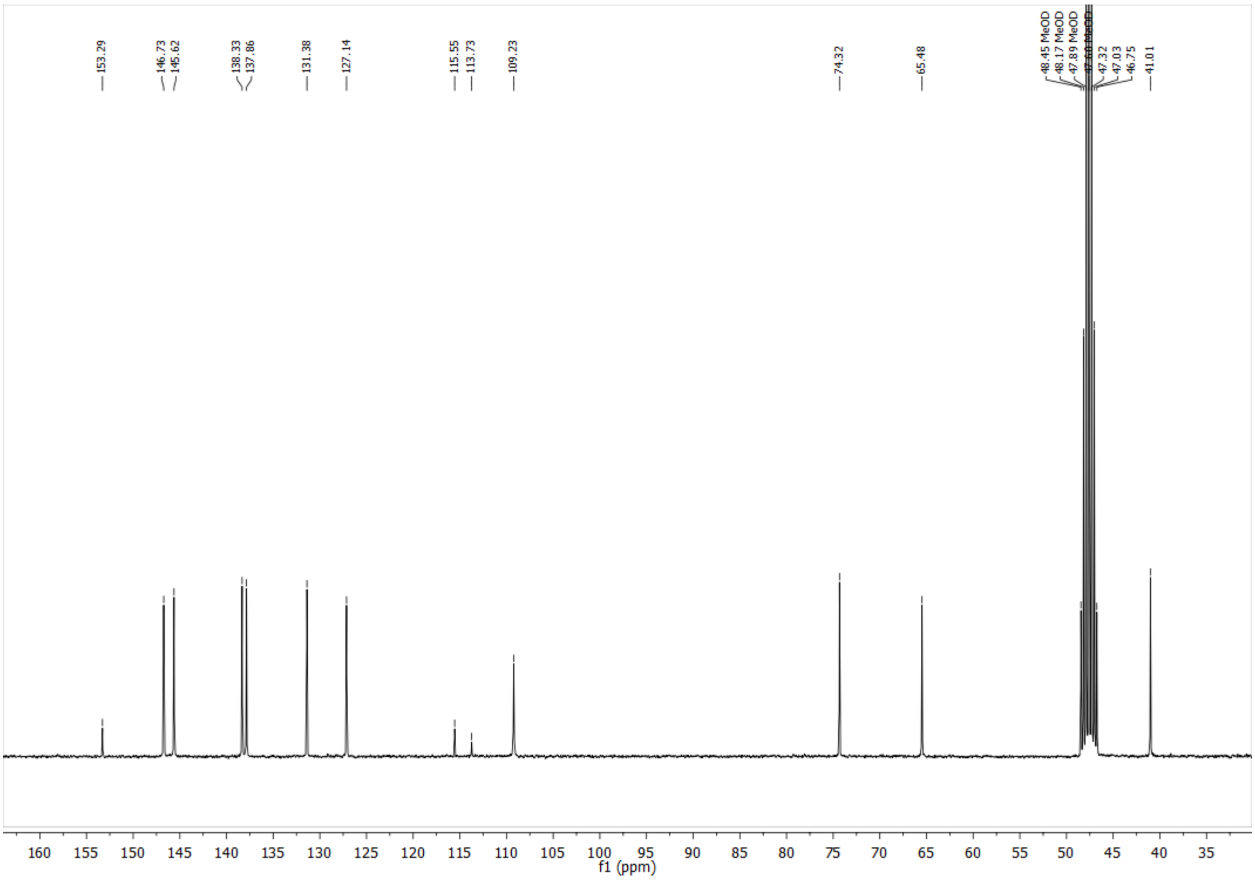

^{1}H-NMR spectrum (300 MHz, $CDCl_3$) of compound **9**

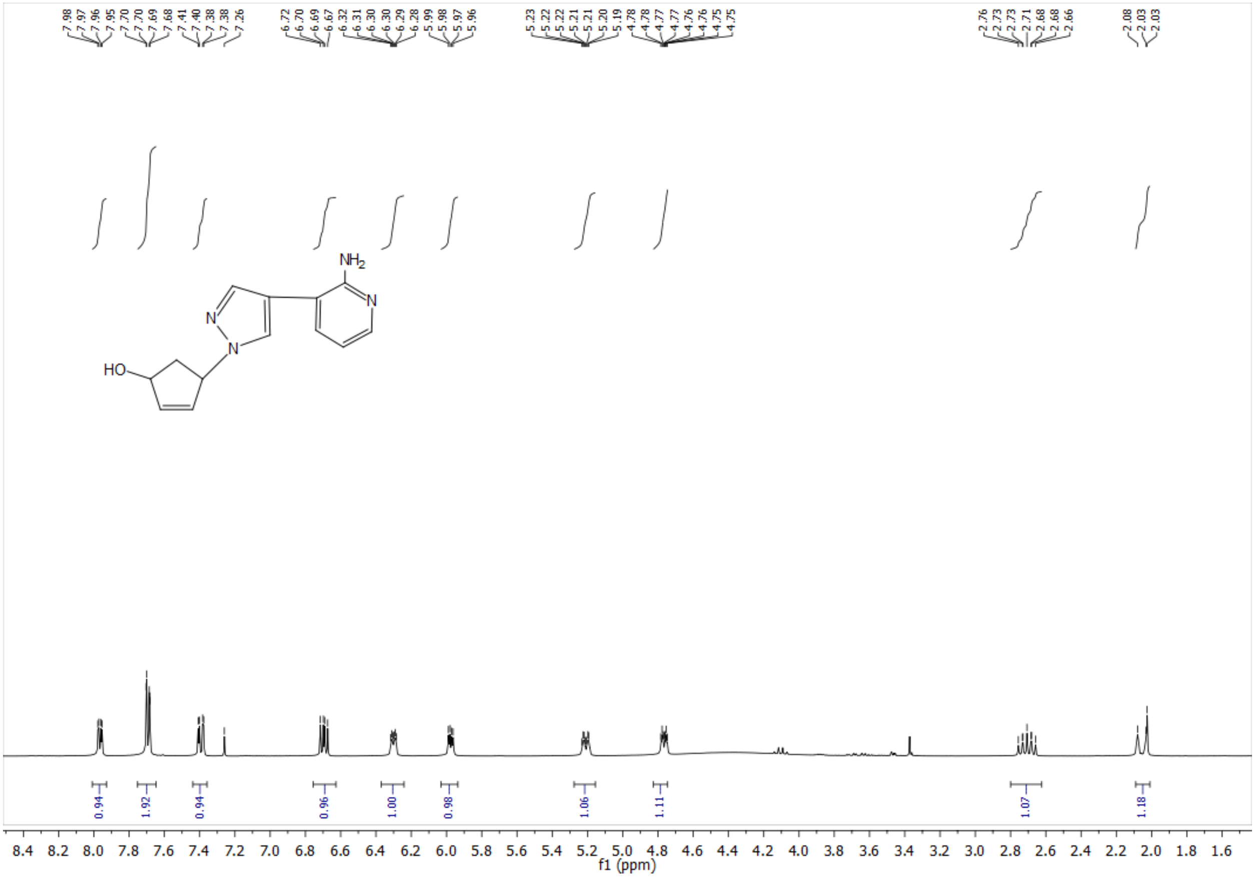

^{13}C-NMR spectrum (75.5 MHz, $CDCl_3$) of compound **9**

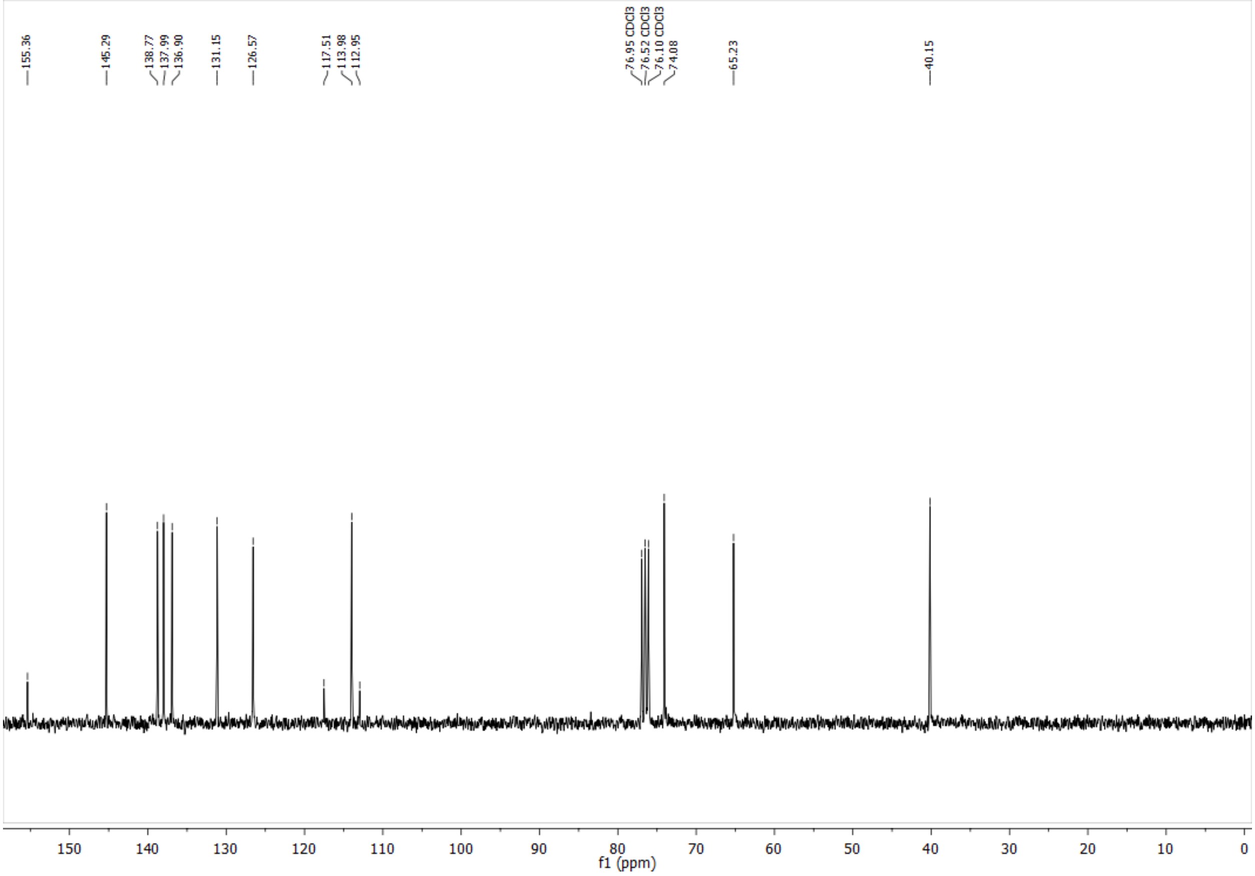

^{1}H-NMR spectrum (300 MHz, CDOD) of compound **10**

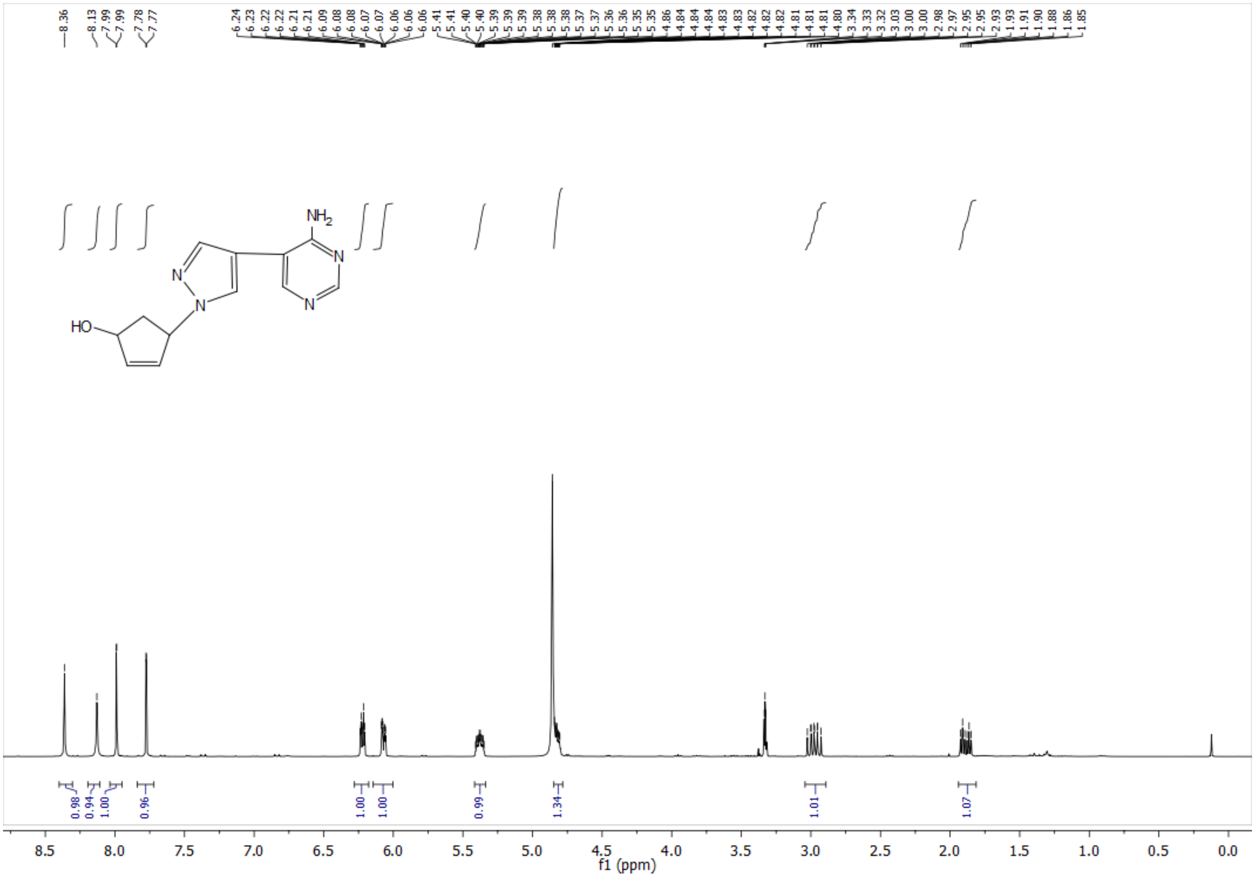

^{13}C-NMR spectrum (75.5 MHz, CDOD) of compound **10**

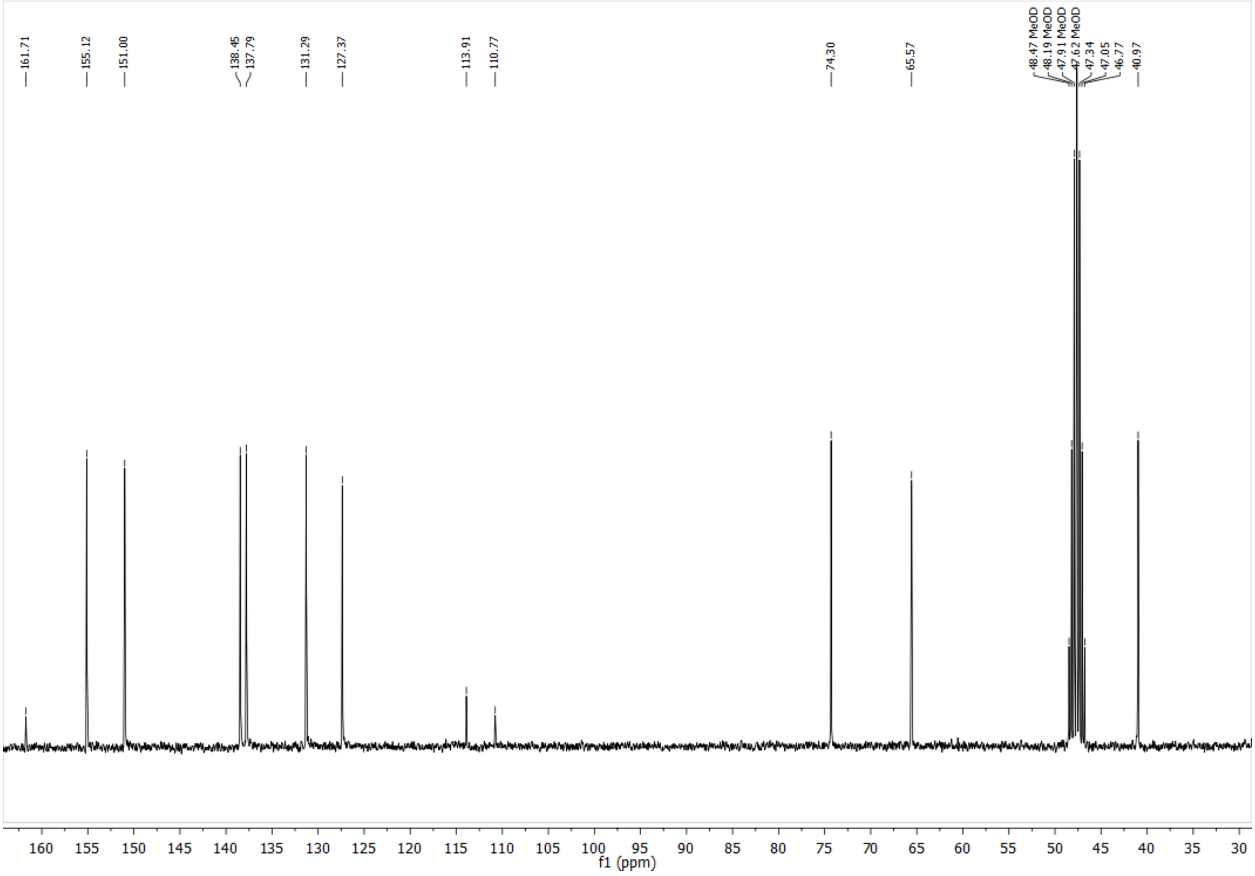

^{1}H-NMR spectrum (300 MHz, CD_3OD) of compound **1**

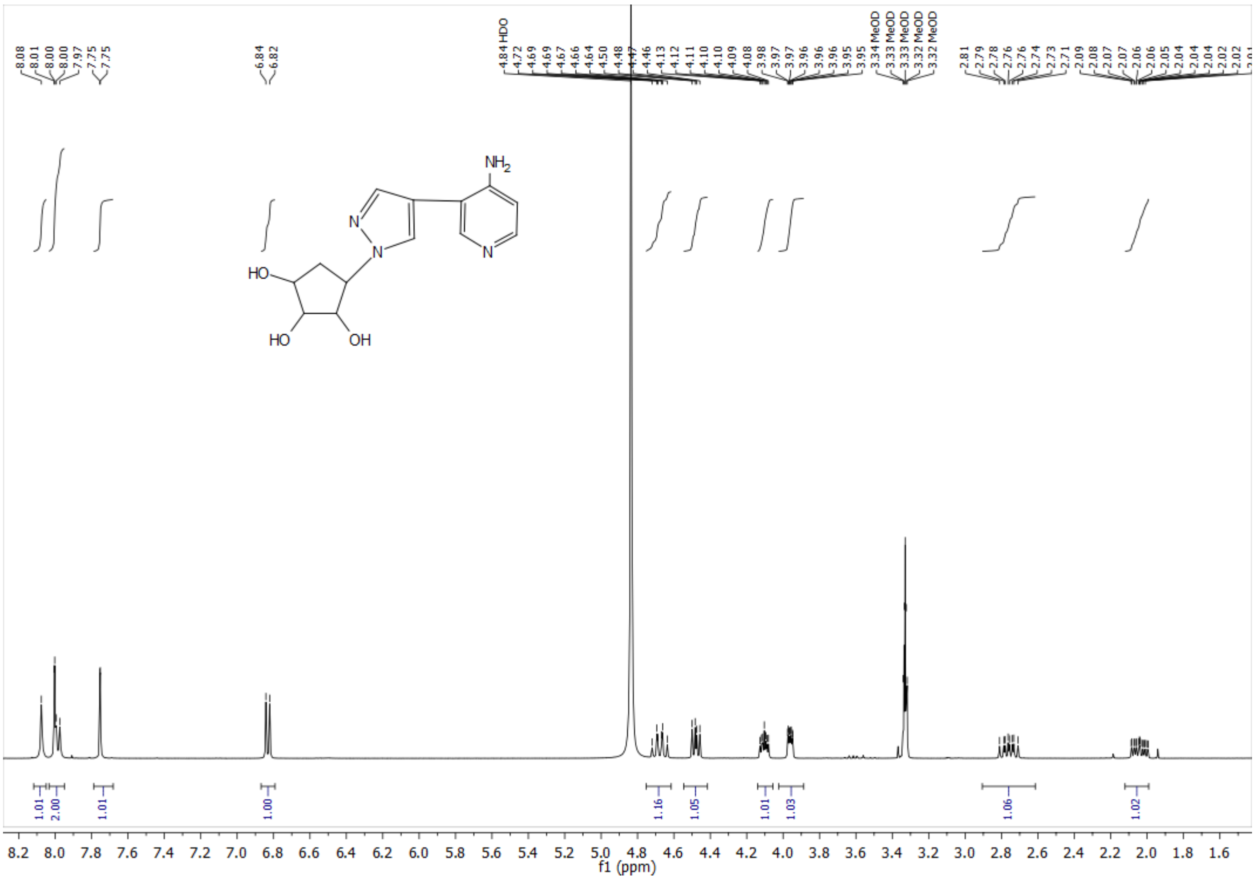

^{13}C-NMR spectrum (75.5 MHz, CD_3OD) of compound **1**

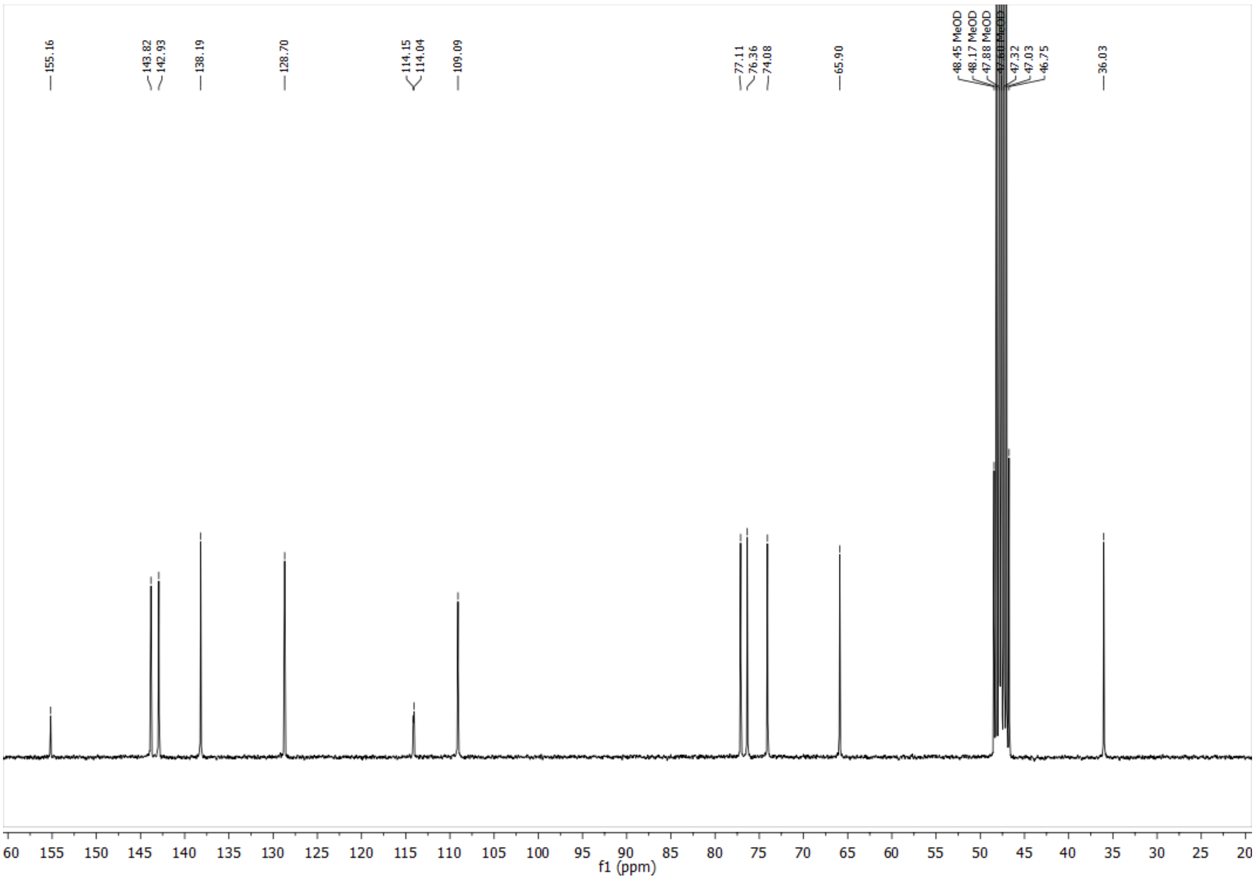

^{1}H-NMR spectrum (300 MHz, CD_3OD) of compound **2**

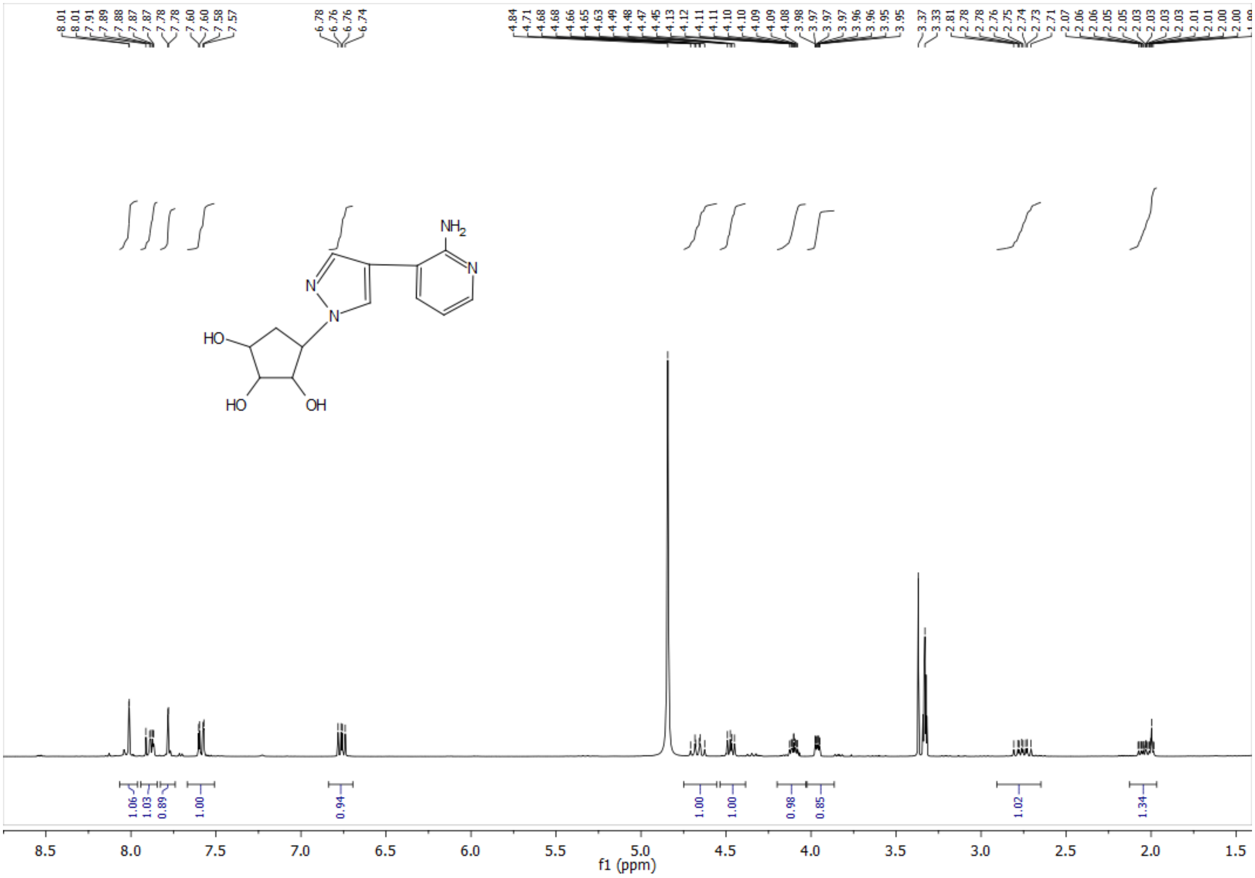

^{13}C-NMR spectrum (75.5 MHz, CD_3OD) of compound **2**

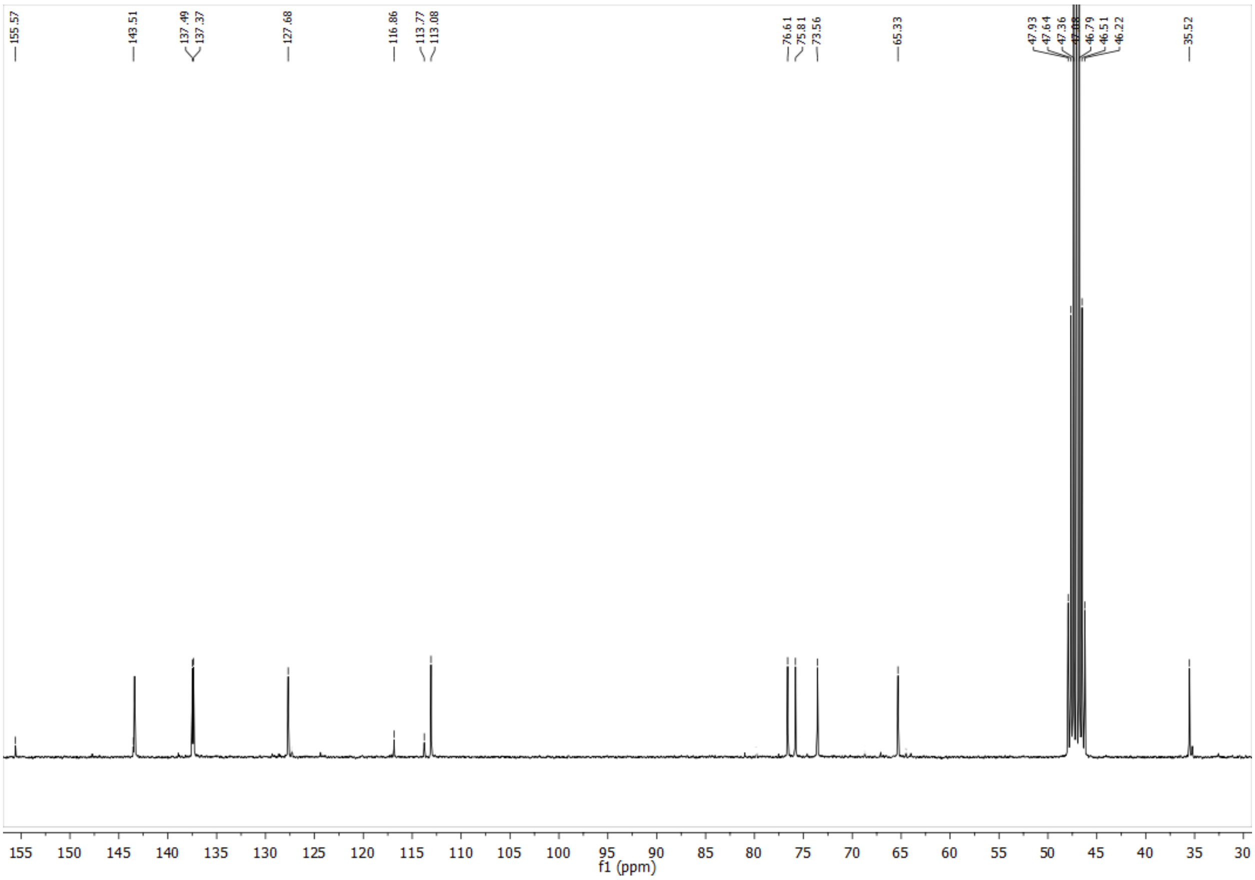

^{1}H-NMR spectrum (300 MHz, CD_3OD) of compound **3**

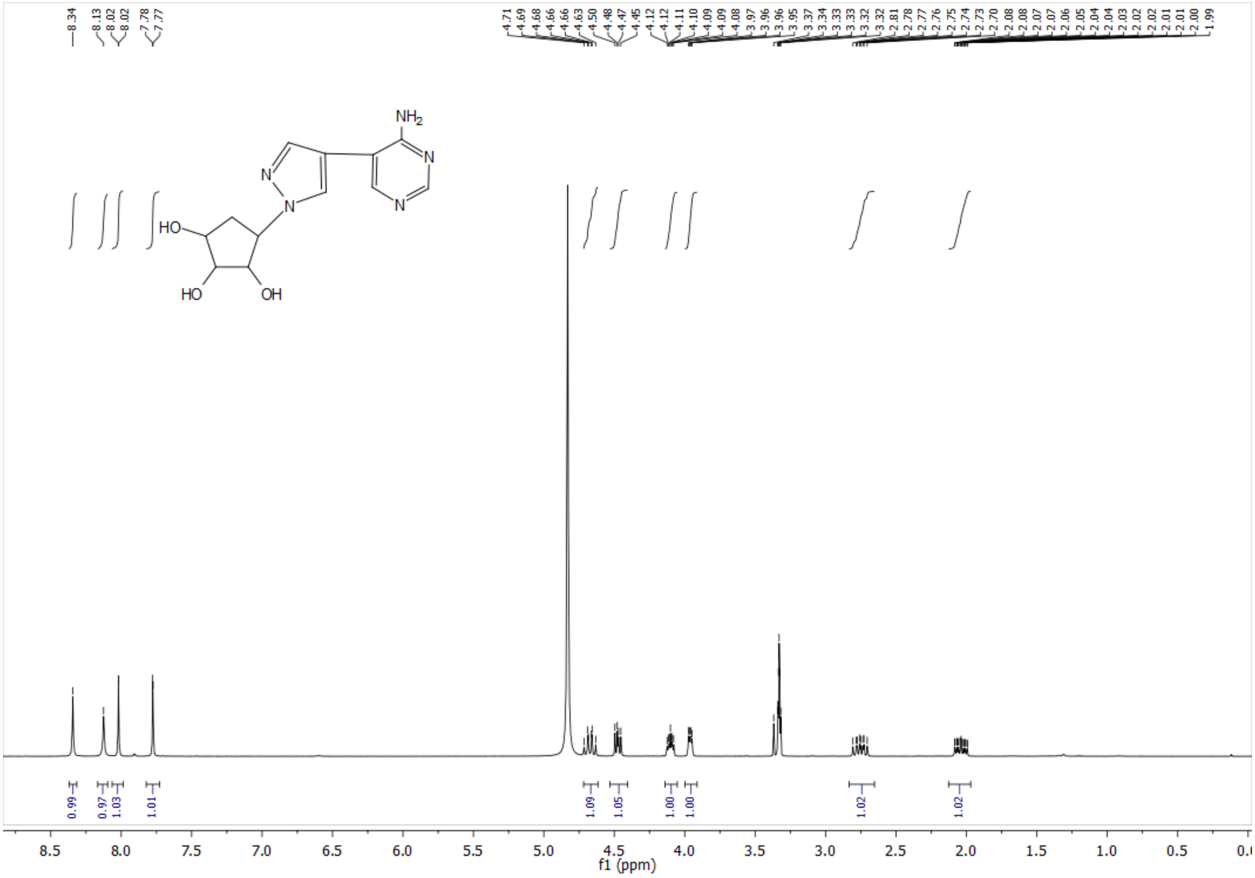

^{13}C-NMR spectrum (75.5 MHz, CD_3OD) of compound **3**

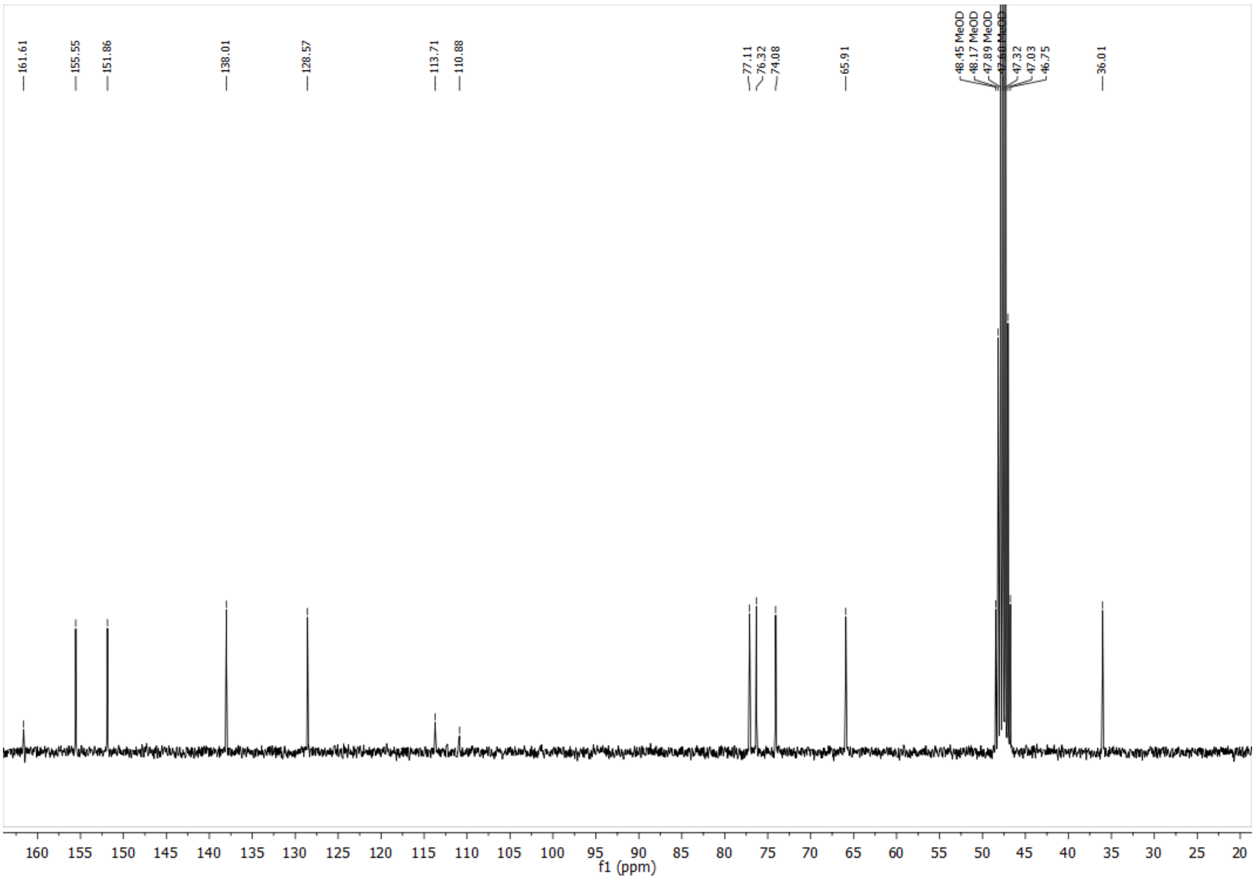

Supplement: Supplementary file 1 [file DataSheet1.pdf]
